# m^6^A demethylase CpALKBH regulates *CpZap1* mRNA stability to modulate the development and virulence of chestnut blight fungus

**DOI:** 10.1128/mbio.01844-24

**Published:** 2024-11-29

**Authors:** Lijiu Zhao, Xiangyu Wei, Fengyue Chen, Baoshan Chen, Ru Li

**Affiliations:** 1 State Key Laboratory for Conservation and Utilization of Subtropical Agro-bioresources, Guangxi Research Center for Microbial and Enzyme Engineering Technology, College of Life Science and Technology, Guangxi University, Nanning, China; 2 Guangxi Key Laboratory of Sugarcane Biology, College of Agriculture, Guangxi University, Nanning, China; Friedrich-Schiller-Universitat, Jena, Germany

**Keywords:** CpALKBH, CpZap1, *Cryphonectria parasitica*, m^6^A demethylase, N^6^-methyladenosine

## Abstract

As the most abundant eukaryotic mRNA modification, N^6^-methyladenosine (m^6^A) plays a crucial role in regulating multiple biological processes. This methylation is regulated by methyltransferases and demethylases. However, the regulatory role and mode of action of m^6^A demethylases in fungi remain poorly understood. In this study, we demonstrate that CpALKBH is a demethylase in *Cryphonectria parasitica* that removes m^6^A modification from single-stranded RNA *in vitro*. The deletion of *CpALKBH* resulted in a significant increase in the m^6^A methylation levels, along with decreases in the growth rate, sporulation, and virulence in *C. parasitica*. Additionally, CpZap1—a transcription factor—was identified as a downstream target of CpALKBH demethylase based on RNA sequencing analysis. We confirmed that CpALKBH demethylase regulates *CpZap1* mRNA stability in an m^6^A-dependent manner. Furthermore, through MazF assay, we found that methylation of *CpZap1* at position 1935A is regulated by both CpALKBH demethylase and CpMTA1 methyltransferase. *CpZap1* significantly influences the fungal phenotype and virulence, thereby restoring the abnormal phenotype observed in ∆*CpALKBH* mutants. Collectively, our findings highlight the essential role of CpALKBH as an m^6^A demethylase in the development and virulence of *C. parasitica*, while also elucidating the molecular mechanisms through which m^6^A modification impacts *CpZap1* mRNA stability.

**IMPORTANCE:**

N^6^-methyladenosine (m^6^A) is the most abundant eukaryotic mRNA modification and is involved in various biological processes. Methyltransferases and demethylases regulate the m^6^A modification, but the regulatory role of m^6^A demethylases in fungi remains poorly understood. Here, we demonstrated that CpALKBH functions as a demethylase in *Cryphonectria parasitica*. The deletion of *CpALKBH* leads to a significant increase in m^6^A levels and a reduction in fungal growth, sporulation, and virulence. We identified *CpZap1* as a downstream target of CpALKBH, with CpALKBH regulating *CpZap1* mRNA stability in an m^6^A-dependent manner. Additionally, our findings indicate that methylation at position 1935A of *CpZap1* is regulated by both the CpALKBH demethylase and the CpMTA1 methyltransferase. Given its critical role in fungal development and virulence, overexpression of *CpZap1* can rescue abnormal phenotypes of ∆*CpALKBH* mutant. Overall, these findings contribute to improving our understanding of the role of m^6^A demethylase in fungi.

## INTRODUCTION

RNA modification, a key mechanism of epigenetic regulation, is widely observed at the transcriptome level. Among these modification, N^6^-methyladenosine (m^6^A) is the most abundant and functionally important internal modification in mRNAs, initially identified in eukaryotic cells in 1974. Other prominent modifications include N6, 2′-O-dimethyladenosine (m^6^Am), N1-methyladenosine (m^1^A), 2′-O-methylation (2′-OMe), and 5-methylcytosine (m^5^C) (1). Specifically, m^6^A refers to the methylation occurring at the N6 position of adenosine. In contrast, m^6^Am is a terminal modification found at the second base next to the 5′ cap in many mRNAs, where it is 2′-O-methylated and further methylated at the N6 position (2). Similar to m^6^A, adding a methyl group to the N1 position of adenosine creates m^1^A modification (3). Among them, m^6^A is the predominant internal modification in eukaryotic mRNA, representing 80% of RNA methylation (4–6). This modification regulates gene expression by various methods, including alternative splicing, localization, transportation, translation, degradation, and stability (7–10). m^6^A modification modulates a wide range of biological processes, such as stem cell differentiation, development in both animals and plants, responses to stress, viral infections, sex determination, cancer progression, and immune responses. Additionally, its significance has been demonstrated in various aspects of plant development, including flowering, trichome development, embryo growth, and fruit ripening (11). Therefore, m^6^A modification holds significant importance in eukaryotes (12, 13).

The dynamic and reversible process of m^6^A modification, involving RNA methyltransferases (writers), demethylases (erasers), and m^6^A-binding proteins (readers) (14), is crucial for post-transcriptional regulation in various kingdoms. The m^6^A demethylases, including fat mass and obesity-associated protein (FTO) demethylase and alpha-ketoglutarate-dependent dioxygenase alkB homolog 5 (ALKBH5) demethylase, catalyze the removal of m^6^A methylation. Both these proteins*—*members of α-ketoglutarate (α-KG)-dependent dioxygenase family*—*facilitate m^6^A demethylation through Fe^2+^ and α-KG-dependent mechanisms (6). FTO demethylase, identified as the first confirmed m^6^A demethylase, catalyzes the demethylation of various substrates. These include m^6^A and cap m^6^Am in mRNA, m^1^A in tRNA, m^6^A and m^6^Am in snRNA (15). However, it has been observed that internal m^6^A modifications in mRNA serve as major substrates for FTO demethylase in various cell types (16). Distinct from FTO demethylase, ALKBH5 is an m^6^A-specific demethylase as it displays no activity toward m^6^Am. Recent reports have found that m^6^A demethylases play critical roles in various developmental processes and human diseases (17). For instance, FTO demethylase contributes to oncogenesis in acute myeloid leukemia, breast cancer, and melanoma via posttranscriptional regulation (18). This role is facilitated by targeting critical transcripts, including ASB2 and RARA (19). In mammals, m^6^A modification can also be affected by the silencing or overexpression of ALKBH5 demethylase. The ALKBH5 was found to be required for mouse fertility, immunity inflammation, and the progression of cancer (17, 20). Additionally, a knockout of *Arabidopsis* m^6^A demethylase ALKBH9B has been found to negatively regulate virus accumulation and systemic invasion, linked to higher m^6^A levels in viral RNA (21).

Contrary to mammals and plants, few studies have explored the role of m^6^A modification in fungi. METTL3 is the primary m^6^A writer that transfers the methyl group from S-Adenosylmethionine (SAM) to N^6^ adenine (22). IME4, a METTL3 homolog in *Saccharomyces cerevisiae* is crucial for triglyceride metabolism, meiosis, mitochondrial impairment, and vacuolar morphology (23). In addition, MTA1*—*the m^6^A writer—in *Pyricularia oryzae* has been found to mediate m^6^A modification and regulate autophagy during fungal infection. PoALKB1 in *P. oryzae* was also reported as an ortholog of human demethylase ALKBH1 (24, 25). Moreover, the functional significance of m^6^A methylation in the biosynthesis of aflatoxin in *Aspergillus flavus* has been investigated. Notably, m^6^A site A332 of the aflatoxin biosynthetic pathway gene *aflQ* has been found to significantly influence aflatoxin production both on culture media and on crop kernels (26). However, recent studies have not sufficiently explored the biological significance of m^6^A demethylase and m^6^A modification in fungal development and virulence.

Here, to advance our knowledge of the role of RNA methylation in phytopathogenic fungi, we focused on *Cryphonectria parasitica*—an important plant pathogen that is destructive to chestnut forests. *C. parasitica* has been widely used as a model filamentous fungus to explore host-virus interactions and fungal pathogenicity due to its ability to support the replication of diverse mycoviruses (27). Various transcriptomic, proteomic, metabolomic, and DNA methylomic studies have unveiled a series of growth- or virulence-related genes (28–32). However, the impact of mRNA methylation/demethylation in the modulation of fungal traits has not been reported in *C. parasitica*.

This study identified a fungal protein exhibiting m^6^A demethylase activity (CpALKBH) in *C. parasitica* and investigated its biological functions by constructing *CpALKBH* deletion mutants. Additionally, we screened putative genes targeted by *CpALKBH* using RNA sequencing (RNA-seq). Furthermore, the association between the transcription factor *CpZap1* mRNA and CpALKBH demethylase was confirmed by RNA immunoprecipitation (RIP). The m^6^A methylation of *CpZap1* was also verified through methylated RNA immunoprecipitation (MeRIP) RT-qPCR and MazF analyses. Moreover, CpMTA1 was found to be the methyltransferase that catalyzes *CpZap1* m^6^A modification. The functional significance of CpZap1 in *C. parasitica* was also elucidated by constructing gene deletion mutant and RNA-seq analysis. In summary, our results offer new insights into the functions and mechanisms of CpALKBH-mediated m^6^A demethylation in filamentous fungi.

## RESULTS

### Characterization of m^6^A demethylase CpALKBH and its role in the development and virulence of *C. parasitica*

**Fig 1:**
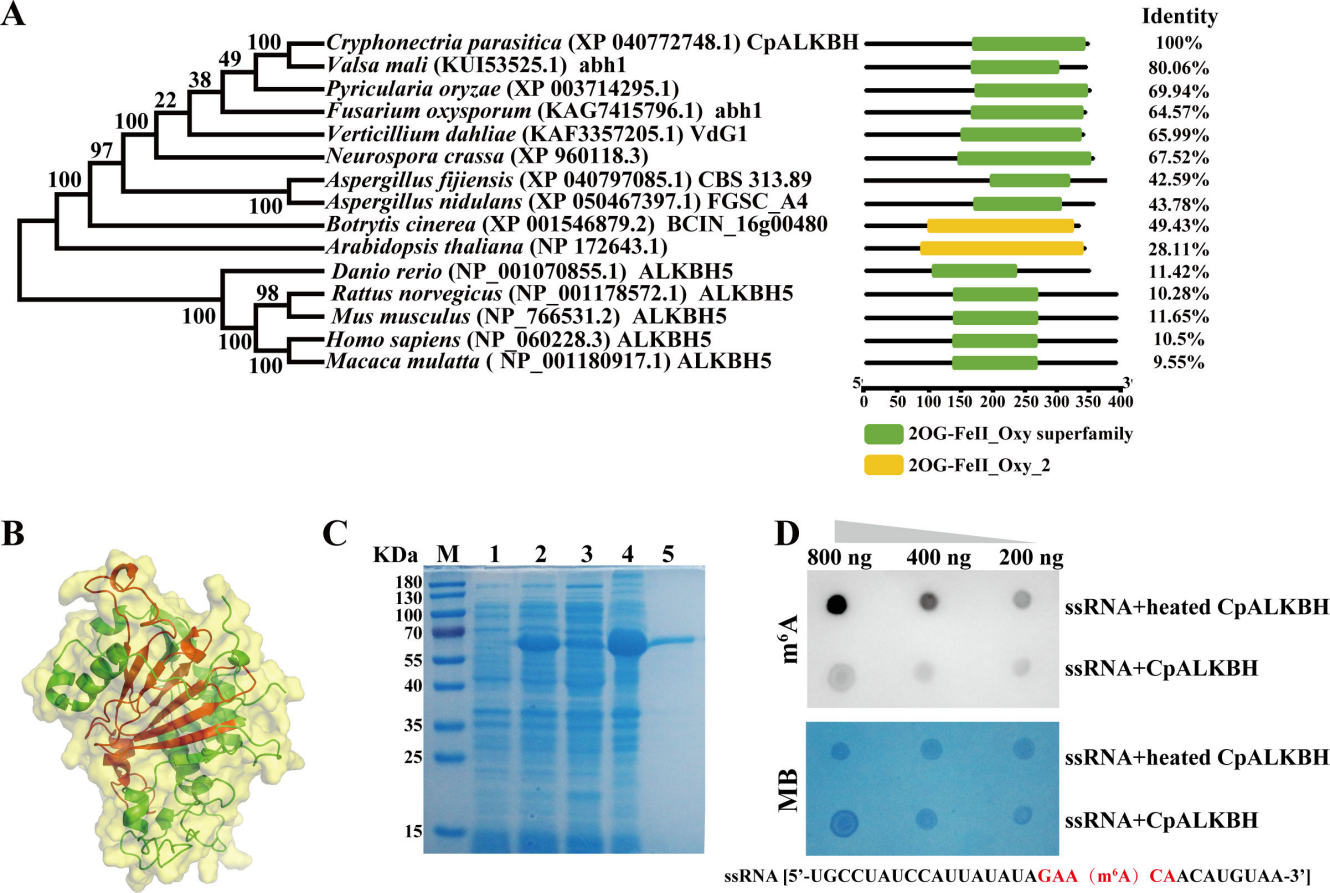
Characterization of the m^6^A demethylase CpALKBH in *C. parasitica*. (**A**) Phylogenetic tree of CpALKBH orthologs from diverse species using MEGAX software analysis. Conserved domain of CpALKBH homologous proteins. The structural domains of these sequences were analyzed using the NCBI website (https://www.ncbi.nlm.nih.gov/Structure/bwrpsb/bwrpsb.cgi) and TBtools software. The sequence similarity between CpALKBH and its homologous proteins was analyzed using DNAMAN software for comparison. (**B**) CpALKBH protein structure prediction using AlphaFold2. The predicted DSBH domain of CpALKBH (222–345 aa) is labeled in red. (**C**) The purified CpALKBH protein was analyzed by SDS-PAGE. M shows molecular mass marker; lane 1 shows uninduced total proteins, lane 2 shows IPTG-induced total proteins, lane 3 shows supernatant proteins; lane 4 shows precipitate proteins; lane 5 shows purified CpALKBH proteins. (**D**) Demethylation of m^6^A in ssRNA by CpALKBH was demonstrated *in vitro* using RNA dot blot assays. The red-marked part represents the m^6^A motif. Different quantities of ssRNA were used for dot blot assay (200 ng, 400 ng, and 800 ng). The heat-inactivated CpALKBH protein did not have enzymatic activity and was used as a control. The loading control was performed by staining with Methylene blue (MB).

To identify the homologous protein of m^6^A demethylase ALKBH5 in the *C. parasitica* genome (33), the amino acid sequence of *Homo sapiens* ALKBH5 (NP_060228.3) was used to perform a BLASTp search against the *C. parasitica* genome (taxid: 660469) available on the NCBI database. This led to the identification of an α-KG-dependent dioxygenase homologous protein (NCBI accession: XP_040772748) in *C. parasitica*—CpALKBH. *CpALKBH* comprises three exons and two introns, encoding a 350-amino acid long protein. Evolutionary phylogenetic relationship analysis showed that the CpALKBH protein is more closely related to homologous proteins of fungi but separated from those of mammals, and it is classified within the ALKBH subfamily of the Fe (II)/2-oxoglutarate (2OG) dioxygenase superfamily (Fig. 1A). The predicted three-dimensional structure of CpALKBH encompasses a highly conserved double-stranded beta-helix fold, a characteristic feature of the α-KG-dependent dioxygenase superfamily and essential for its catalytic core function (34) (Fig. 1B). Furthermore, we found that the purified CpALKBH protein could remove m^6^A modification of the synthesized RNA probe (containing a GAACA motif) (Fig. 1C and D). This indicates that CpALKBH is a protein identified in *C. parasitica* as RNA m^6^A demethylase activity.

**Fig 2:**
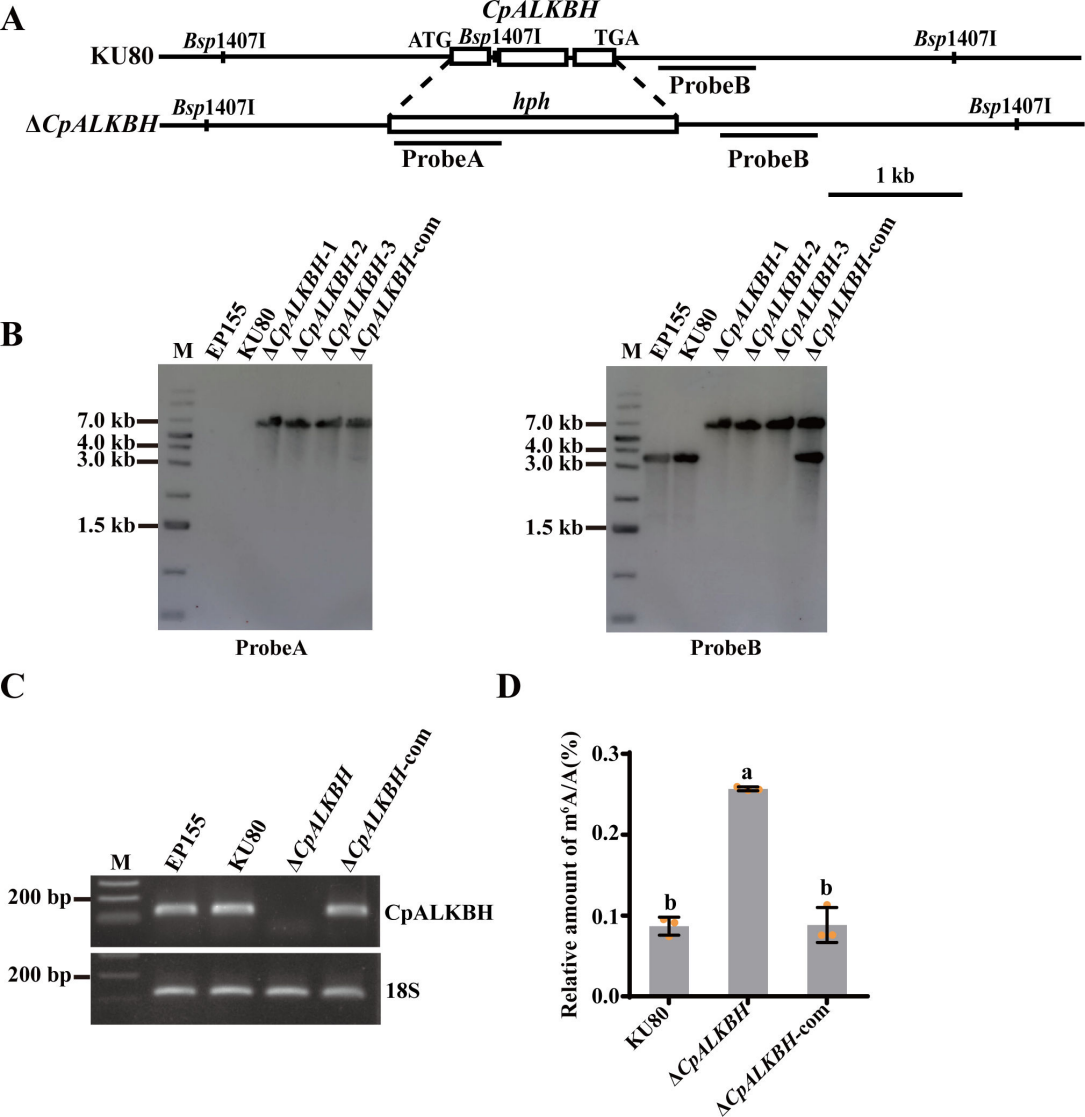
The deletion of *CpALKBH* alters the m^6^A RNA methylation level in *C. parasitica*. (**A**) *CpALKBH* gene deletion strategy in schematic form. Southern blotting used a fragment from the probe A (*hph* gene) and the probe B (a fragment from the right arm) to compare the fragment sizes between the KU80 strain and *CpALKBH* deletion mutants. The scale bar represents 1 kb. (**B**) Using probe A and B, Southern blotting analysis was conducted on Δ*CpALKBH* mutants and KU80. Genomic DNA from fungi was digested with *Bsp*1407I, electrophoresed on agarose gels, then probed with probe A and probe B. The strains used were EP155 (wild-type), KU80 (parental strain), ∆*CpALKBH* (*CpALKBH* deletion strain), and ∆*CpALKBH*-com (complementary strain). EP155 and KU80 were used as control. (**C**) The deletion of the *CpALKBH* gene was verified using RT-PCR, with the 18S rRNA gene serving as an internal control. (**D**) Methylation level of m^6^A RNA was determined with an RNA methylation detection kit. A standard deviation from three independent experiments is shown with the error bars. Treatment differences are indicated by different letters above the bars. (ANOVA followed by Tukey’s test, *P* < 0.05).

To explore the biological role of *CpALKBH* in *C. parasitica*, the *CpALKBH* deletion mutant was generated by replacing the *CpALKBH* gene with a hygromycin-resistant gene. The single-spored Δ*CpALKBH* mutants were verified through PCR, qRT-PCR, and Southern blotting analysis. To ensure that the phenotypic changes were actually caused by the deletion of *CpALKBH*, we complemented Δ*CpALKBH* mutants *in trans* with a wild-type allele of *CpALKBH* (Fig. 2A through C; Fig. S1).

To examine whether *CpALKBH* is responsible for RNA m^6^A demethylation in *C. parasitica*, global m^6^A methylation levels were quantified by ELISA in all the RNA samples extracted from the control strain KU80, Δ*CpALKBH*, and Δ*CpALKBH*-com. In the Δ*CpALKBH* mutant, the m^6^A RNA content reached 0.256% ± 0.001% of the total RNA, representing a 1.95-fold increase compared to the KU80 strain (0.087% ± 0.006% of the total RNA), highlighting a marked rise in m^6^A modification. The m^6^A modification level returned to normal in the complemented strain Δ*CpALKBH*-com (Fig. 2D). These results demonstrate that *CpALKBH* is involved in the m^6^A demethylation of *C. parasitica*.

**Fig 3:**
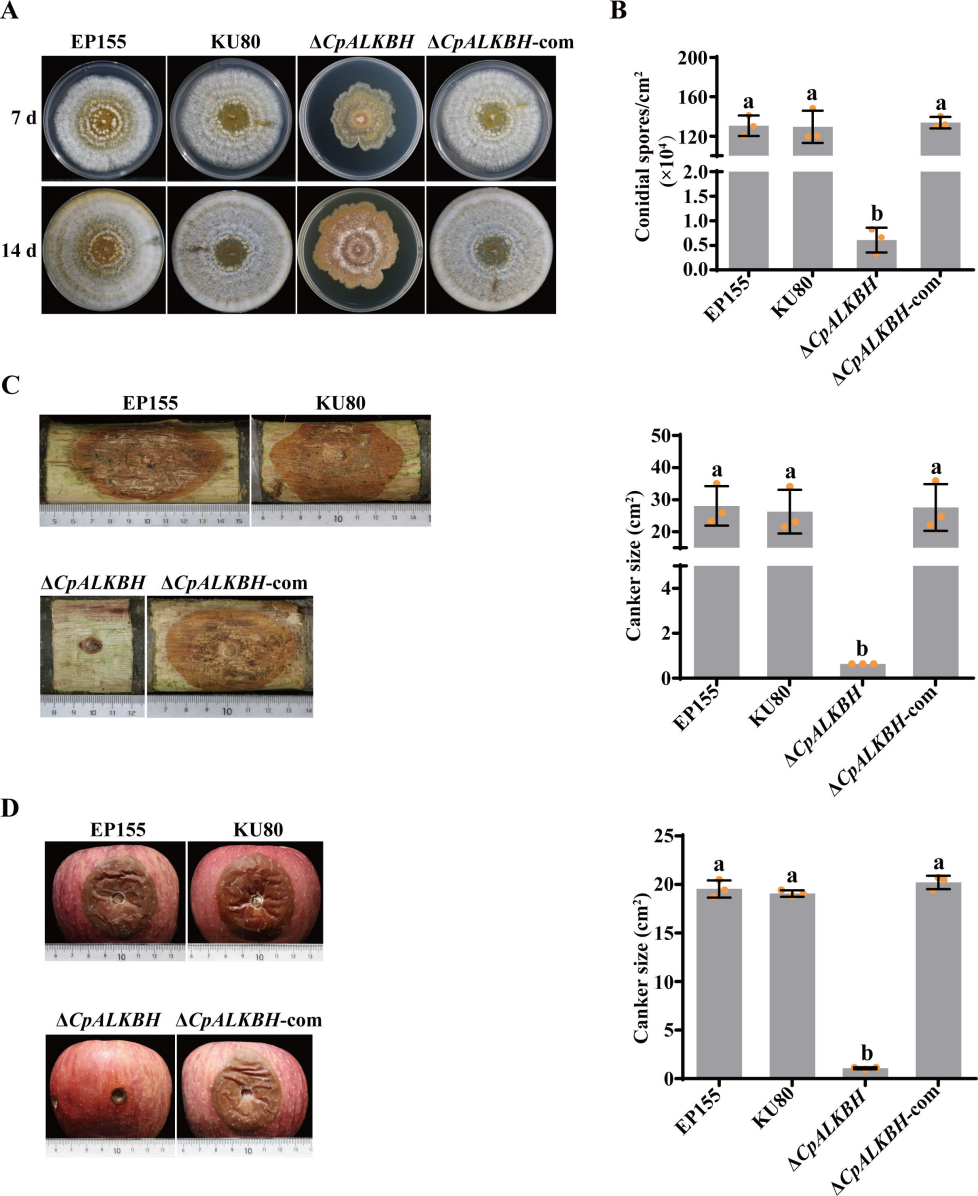
Analysis of phenotype, sporulation, and virulence of Δ*CpALKBH* mutant strains. (**A**) Photographs of mutant colonies were taken at 7 and 14 days post-inoculation to assess colony morphology. The strains displayed included EP155, KU80, Δ*CpALKBH,* and ∆*CpALKBH*-com. (**B**) The sporulation levels of the indicated strains were measured on day 14. (**C**) The dormant Chinese chestnut stems were inoculated with the tested strains and kept at 26°C for 25 days. (**D**) The Red Fuji apples were inoculated with the tested strains at 26°C for 10  days. A standard deviation from three independent experiments is shown with the error bars. Treatment differences are indicated by different letters above the bars. (ANOVA followed by Tukey’s test, *P* < 0.05).

To investigate the function of CpALKBH, the growth patterns of mutants were compared with the wild-type strains. The growth rate of Δ*CpALKBH* was slower than that of EP155 and KU80 strains, and its colony margin was irregular (Fig. 3A). Additionally, the *CpALKBH* deletion significantly reduced sporulation in the mutant compared to the wild-type strain (Fig. 3B). Furthermore, the abnormal phenotype of the knockout mutant was fully restored in the complementation strain.

To further investigate whether *CpALKBH* is essential for the virulence of *C. parasitica*, chestnut stems and red Fuji apples were used. The hypovirulent strain EP155/CHV1-EP713 was used as the control. Compared to that of EP155 and KU80 strains, the virulence of ∆*CpALKBH* was significantly decreased. The complemented strain ∆*CpALKBH*-com restored the mutant virulence to a level comparable to that of the parent strain (Fig. 3C and D). The findings collectively substantiate the critical role of CpALKBH in fungal development and virulence.

### Transcriptomic analysis reveals the regulatory function of CpALKBH

**Fig 4:**
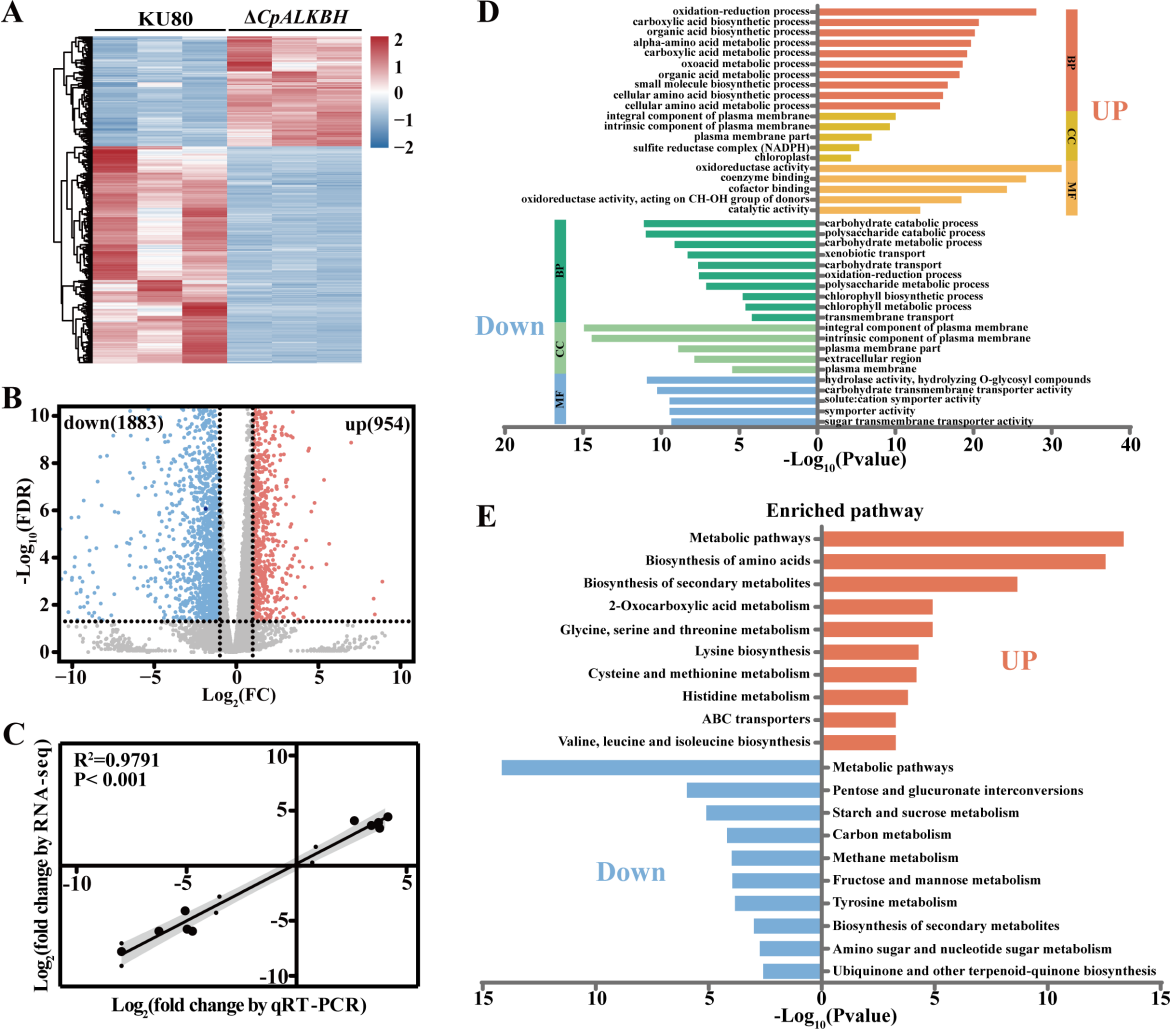
RNA-seq analysis identified transcripts affected by *CpALKBH*. (**A**) RNA-seq heatmap showing relative gene expression in the Δ*CpALKBH* compared to the KU80. (**B**) The DEGs between the KU80 and Δ*CpALKBH* strains were shown with a Volcano plot. mRNAs log_2_FC > 1 in Δ*CpALKBH* relative to KU80 (*P* value < 0.01) were highlighted in red. mRNAs log_2_FC < −1 in Δ*CpALKBH* relative to KU80 (*P* value < 0.01) were highlighted in blue. (**C**) The expression of 10 randomly chosen DEGs was validated through RNA-seq and qRT-PCR analysis. From three biological samples, Log_2_FC values and the coefficient of determination (***R*^2^**) were calculated. (**D**) DEG enrichment analysis based on biological process (BP), cellular component (CC), and molecular function (MF). (**E**) Analyses of DEGs enriched for KEGG pathways. DEG, differentially expressed gene.

To further elucidate the regulatory role of *CpALKBH*, the mycelial samples of KU80 and Δ*CpALKBH* strains were collected 7 days post-inoculation for transcriptome sequencing (RNA-seq). A total of 37.5–43.8 million and 43.3–48.9 million reads were obtained for KU80 and Δ*CpALKBH* strains, respectively. After removing adapter sequences and low-quality reads, 37.3–43.5 million and 43.1–48.7 million reads were used for further analysis (Table S1). Hierarchical clustering of RNA-seq data revealed notable differences in the mRNA expression heat maps between the KU80 and Δ*CpALKBH* groups (Fig. 4A). RNA-seq database identified 954 upregulated genes and 1,883 downregulated genes in Δ*CpALKBH* compared to KU80 strain [log_2_(fold change) >1, *P* < 0.05] (Fig. 4B; Table S2). The reliability of the RNA-seq data was verified using qRT-PCR analysis of 10 randomly selected DEGs. A strong correlation (correlation coefficient *R*^2^ = 0.9791, *P* < 0.001) between RNA-seq and qRT-PCR results further confirmed their consistency (Fig. 4C; Table S2). As shown in Fig. 4D and E, these DEGs were subjected to gene ontology (GO) enrichment and Kyoto Encyclopedia of Genes and Genomes (KEGG) pathway analysis. Specifically, the DEGs downregulated in the Δ*CpALKBH* group were significantly associated with KEGG pathways such as pentose and glucuronate interconversions, starch and sucrose metabolism, methane metabolism, amino sugar and nucleotide sugar metabolism, and GO terms like polysaccharide catabolic process and extracellular region. In comparison to the KU80 strain, the upregulated genes in the Δ*CpALKBH* group were primarily associated with amino acid biosynthesis, 2-oxocarboxylic acid metabolism, and ABC transporters (KEGG), as well as alpha-amino acid metabolism, small-molecule biosynthesis, and the sulfite reductase complex (NADPH) (GO).

### CpALKBH regulates the stability of *CpZap1* mRNA through an m^6^A-dependent manner

**Fig 5:**
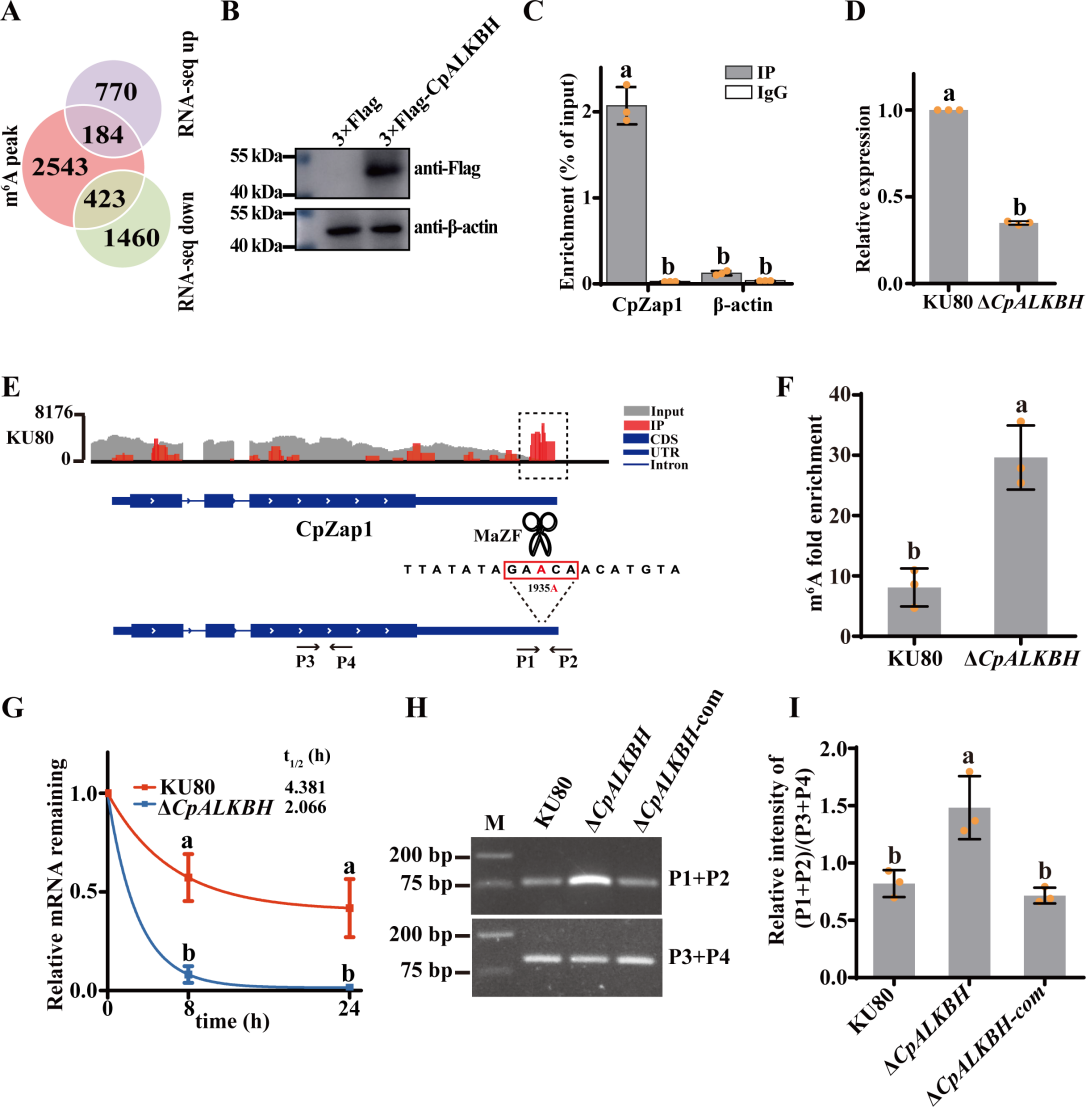
CpALKBH regulates *CpZap1* mRNA stability in an m^6^A-dependent manner. (**A**) Venn diagram illustrating the number of overlapping genes between the m^6^A-seq and RNA-seq data. (**B**) The EP155/3 × Flag-CpALKBH strain was verified using Western blotting with an anti-Flag antibody and anti-β-actin antibody. (**C**) RIP assay demonstrated the interaction between *CpZap1* mRNA and CpALKBH protein. It was performed with an anti-Flag antibody in the EP155/3 × Flag-CpALKBH strain. Negative controls were made with β-actin. Normalizing fold enrichment values to input was done. (**D**) qRT-PCR analysis confirmed *CpZap1* expression levels in KU80 and Δ*CpALKBH*. (**E**) Integrative genomics viewer (IGV) plots show m^6^A peaks on the *CpZap1* transcript in KU80, with the *Y*-axis representing normalized read counts. A schematic of the MazF enzyme assay was also included, with scissors indicating the MazF; the MazF ACA site within the methylation site is highlighted in red (bottom). (**F**) MeRIP-qRT-PCR analysis with anti-m^6^A antibody was conducted to detect m^6^A levels of *CpZap1* in KU80 and Δ*CpALKBH* mutant. (**G**) *CpZap1* expression levels were measured by qRT-PCR in KU80 and CpALKBH after actinomycin D treatment, and the mRNA half-life (*t*_1/2_) of *CpZap1* was assessed using a nonlinear regression model. (**H**) PCR amplification was performed on cDNA from MazF-digested mRNA. The positions of primers P1 and P2 relative to the targeted methylation site are illustrated in E. Control primers P3 and P4, which do not flank an ACA site, were also utilized. (**I**) The result of (**H**) was analyzed using ImageJ. The relative intensity of the (P1 + P2)/(P3 + P4) was calculated. The product level of strains with primers P1 + P2 in KU80, Δ*CpALKBH,* and Δ*CpALKBH*-com was normalized with the control P3 + P4, respectively. A standard deviation from three independent experiments is shown with the error bars. Treatment differences are indicated by different letters above the bars (ANOVA followed by Tukey’s test, *P* < 0.05).

To identify the downstream targets of CpALKBH effectively, we overlapped the above RNA-seq data with previously obtained m^6^A-seq results of the KU80 strain (accession no. SRP475797). The m^6^A-seq identified 4510 m^6^A peaks from 3150 m^6^A-modified genes in the KU80 strain. By overlapping m^6^A-modified genes and DEGs, we identified 184 upregulated genes and 423 downregulated genes with m^6^A modification, which are potential CpALKBH targets (Fig. 5A). Considering that transcription factors are regulated by RNA demethylases in an m^6^A-dependent manner (35), we conducted a screening of 423 downregulated genes and identified a single transcription factor, CpZap1, for further investigation (Table S3). CpZap1 was annotated with a classical C2H2 zinc finger domain (XP_040772497.1) through the NCBI database, which was well conserved among different organisms (Fig. S2). C2H2 transcription factors are a major family of fungal zinc finger regulators found in all eukaryotes. They primarily regulate fungal development, stress tolerance, and metabolism in plant-pathogenic fungi (36). To assess whether CpZap1 has transactivation activity, the entire *CpZap1* cDNA was cloned into a pGBKT7 vector and introduced into Y2HGold yeast cells. Yeast cells transformed with the fusion plasmid (pGBKT7-*CpZap1*) or positive control grew on the SD/-Trp/-Leu/-His/-Ade medium, whereas yeast cells transformed with the negative control did not grow (Fig. S3A). Additionally, subcellular localization showed that *CpZap1* was located in the nucleus (Fig. S3B). These findings indicate that *CpZap1* acts as a transcription factor.

To examine the interaction between CpALKBH and *CpZap1* transcripts, RIP of the EP155/3 × Flag-CpALKBH strain was performed. Fig. 5B and C showed that *CpZap1* mRNA was significantly enriched with anti‐Flag antibody. This revealed that *CpZap1* mRNA was a direct target of CpALKBH. Furthermore, the significantly downregulated expression of *CpZap1* in Δ*CpALKBH* was confirmed by qRT-PCR (Fig. 5D). As CpALKBH is an m^6^A demethylase, it was presumed to regulate the expression of *CpZap1* by removing its m^6^A modification. The KU80 m^6^A-seq findings revealed a significant m^6^A peak (chr8:1334739–1334863) distributed in the 3'UTR region of *CpZap1* mRNA (Fig. 5E). MeRIP was then performed in combination with *CpZap1*-specific qRT-PCR to detect the changes in *CpZap1* m^6^A modification following *CpALKBH* deletion. The findings verified that m^6^A methylation was readily detectable on *CpZap1* mRNA in the KU80 strain. *CpALKBH* deletion further increased the m^6^A level in *CpZap1* mRNA compared with that in the control (Fig. 5F), thereby confirming the role of *CpALKBH* as the m^6^A demethylase responsible for *CpZap1* demethylation. To further evaluate the stability of *CpZap1* mRNA after *CpALKBH* deletion, an RNA decay assay was performed by the transcription inhibitor actinomycin D in KU80 and Δ*CpALKBH* strains. As illustrated in Fig. 5G, the deletion of *CpALKBH* markedly reduced the half-life of *CpZap1* mRNA, thereby indicating its impact on *CpZap1* mRNA stability.

To determine the specific methylation sites on *CpZap1* transcripts, we employed the MazF RNA restriction enzyme. It cleaves RNA at ACA sites but not m^6^ACA sites in a methylation-sensitive manner (37). The ACA sequence is a part of the GAm^6^ACA sequence of *CpZap1* m^6^A peaks shown in Fig. 5E. Through motif screening analysis, 1935A at 3′UTR was speculated to be a key methylation modification site. The purified mRNA isolated from the KU80, Δ*CpALKBH,* and Δ*CpALKBH*-com strains was subjected to digestion with MazF, followed by reverse transcription to generate cDNA. To exclude the possibility of genomic DNA (gDNA) contamination, primers targeting the *CpZap1* intron were also designed for the assay (Fig. S4A). The cDNA, thus, obtained was amplified using primers P1/P2, resulting in the generation of corresponding products. The cDNA yields from Δ*CpALKBH* were significantly higher than those from the KU80 and Δ*CpMTA1*-com strains, as demonstrated in Fig. 5H and I. Meanwhile, PCR with primers P3/P4 was used as a negative control, as this region did not contain a potential MazF cleavage site. This result suggests that *CpZap1* undergoes methylation at the 1935A position—a process regulated by the demethylase CpALKBH. Taken together, these observations collectively support that CpALKBH mediates *CpZap1* mRNA stability in an m^6^A‐dependent manner.

### CpMTA1 catalyzes m^6^A modification of *CpZap1*

**Fig 6:**
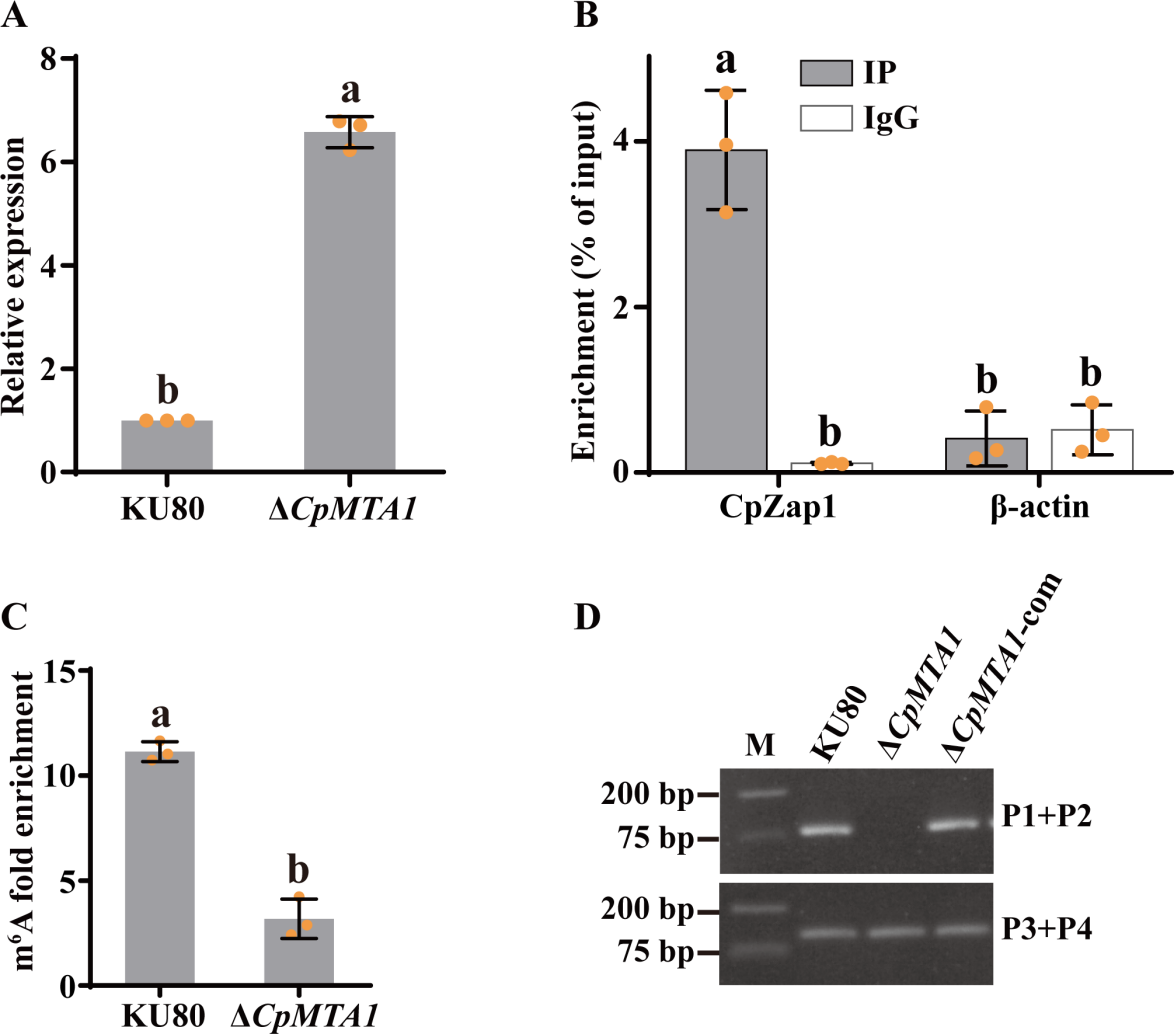
CpMTA1 catalyzes the m^6^A modification of *CpZap1*. (**A**) qRT-PCR analysis confirming *CpZap1* expression levels in KU80 and Δ*CpMTA1*. (**B**) RIP assay demonstrated the interaction between *CpZap1* mRNA and CpMTA1 protein. It was performed with an anti-Flag antibody in the EP155/3 × Flag-CpMTA1 strain. Negative controls were made with β-actin. Normalizing fold enrichment values to input was done. (**C**) MeRIP-qPCR analysis was conducted to assess m^6^A levels of *CpZap1*. mRNA from the KU80 and Δ*CpMTA1* mutant strains was used for m^6^A-IP, employing an anti-m^6^A antibody. The resulting IP products served as templates for the subsequent MeRIP-qPCR. (**D**) PCR amplification was performed on cDNA synthesized from MazF-digested mRNA. A standard deviation from three independent experiments is shown with the error bars. Treatment differences are indicated by different letters above the bars (ANOVA followed by Tukey’s test, *P* < 0.05).

The dynamic and reversible characteristics of m^6^A modification in eukaryotic cells has already been established (38). In a previous study, we identified that CpMTA1 is an m^6^A methyltransferase in *C. parasitica* (39). Therefore, we investigated the potential role of CpMTA1 in catalyzing the m^6^A modification of *CpZap1*. Initially, we assessed the impact of *CpMTA1* deletion on the expression level of *CpZap1* and observed 6.5-fold increase in the expression level in Δ*CpMTA1* compared to that in the KU80 strain (Fig. 6A). Furthermore, RIP assay revealed a direct interaction between *CpZap1* mRNA and CpMTA1 in the EP155/3 × Flag-CpMTA1 strain (Fig. 6B). These findings indicate that *CpZap1* may be a substrate for CpMTA1 methyltransferase. To verify this, MeRIP combined with qRT-PCR was performed to analyze the levels of *CpZap1* m^6^A methylation following *CpMTA1* deletion. The findings revealed a small *CpZap1* m^6^A peak indicating significantly decreased methylation levels in Δ*CpMTA1* mutant compared to that in the KU80 strain, confirming that CpMTA1 functions as the methyltransferase responsible for catalyzing the m^6^A modification of *CpZap1* (Fig. 6C).

Furthermore, we verified that the deletion of *CpMTA1* resulted in a decreased m^6^A level at the 1935A site of *CpZap1* mRNA. The purified mRNA from the KU80, Δ*CpMTA1,* and Δ*CpMTA1*-com strains was digested by MazF, by reverse transcription to synthesize cDNA. No contamination of gDNA was confirmed (Fig. S4B). PCR amplification of the resulting cDNA with primers P1/P2 yielded a corresponding product with cDNA derived from the KU80 and Δ*CpMTA1*-com strains but not from the Δ*CpMTA1* strain (Fig. 6D). The findings showed that the 1935A site of *CpZap1* was protected from MazF cleavage in mRNA from strains containing a functional methyltransferase, indicating that CpMTA1 could catalyze *CpZap1* mRNA methylation at the 1935A site *in vivo*.

### The regulatory impact of CpZap1 on *C. parasitica* development, virulence, and transcriptional activity

**Fig 7:**
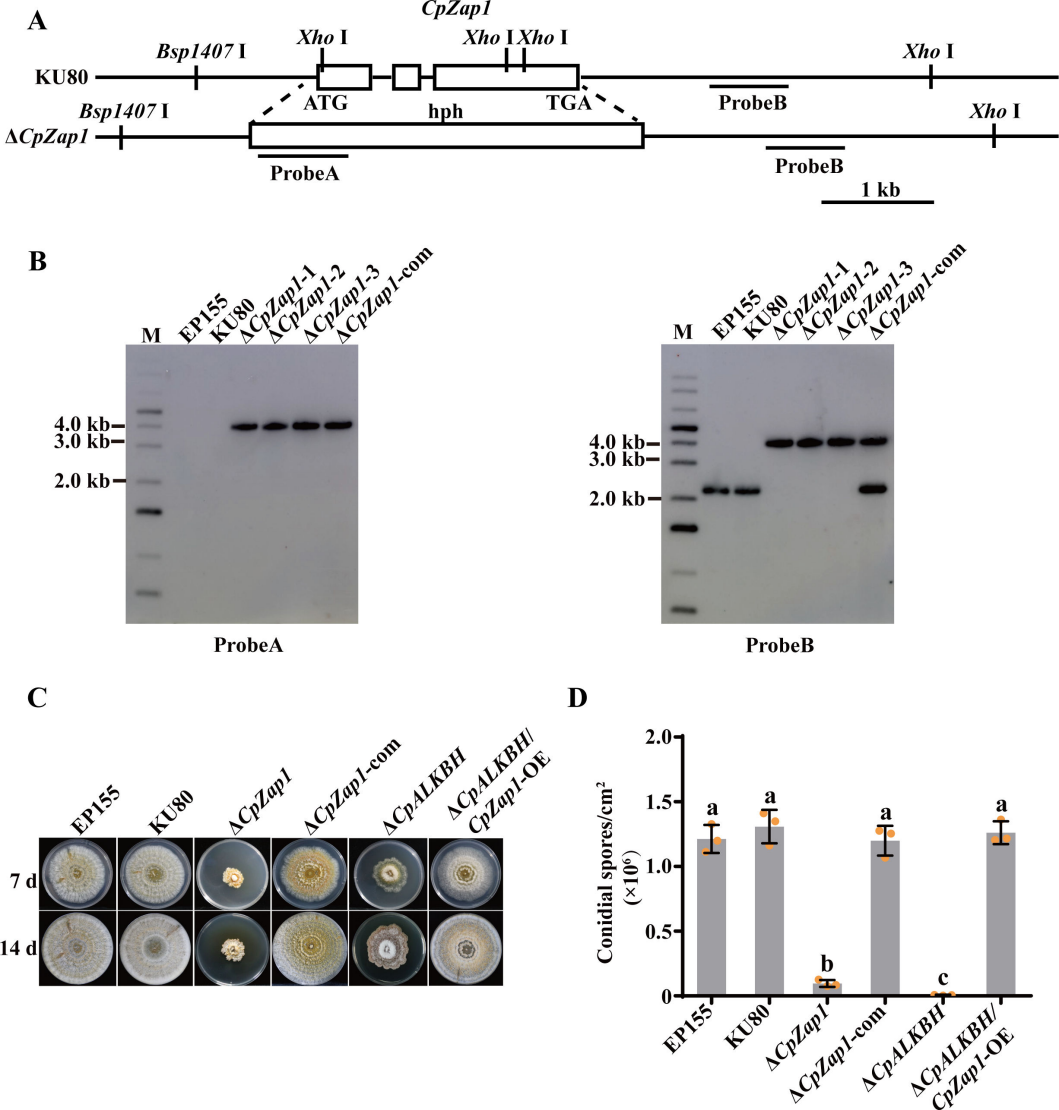
Construction and phenotype analysis of *CpZap1* mutant strains. (**A**) *CpZap1* gene deletion strategy in schematic form. Southern blotting used a fragment from the probe A (*hph* gene) and the probe B (a fragment from the right arm) to compare the fragment sizes between the KU80 strain and *CpZap1* deletion mutants. The scale bar represents 1 kb. (**B**) Using probe A and B, Southern blotting analysis was conducted on Δ*CpZap1* mutants and KU80. Genomic DNA from fungi was digested with *Bsp*1407I and *Xho* I, electrophoresed on agarose gels, and then probed with probe A and probe B. The strains used were EP155 (wild-type), KU80 (parental strain), ∆*CpZap1* (*CpZap1* deletion strain), and ∆*CpZap1*-com (complementary strain). EP155 and KU80 were used as control. (**C**) Photographs of mutant colonies were taken at 7 and 14 days post-inoculation to assess colony morphology. The strains displayed included EP155, KU80, ∆*CpZap1*, ∆*CpZap1*-com, and ∆*CpALKBH*/*CpZap1*-OE. (**D**) Sporulation levels of the tested strains were assessed on day 14. A standard deviation from three independent experiments is shown with the error bars. Treatment differences are indicated by different letters above the bars (ANOVA followed by Tukey’s test, *P* < 0.05).

**Fig 8:**
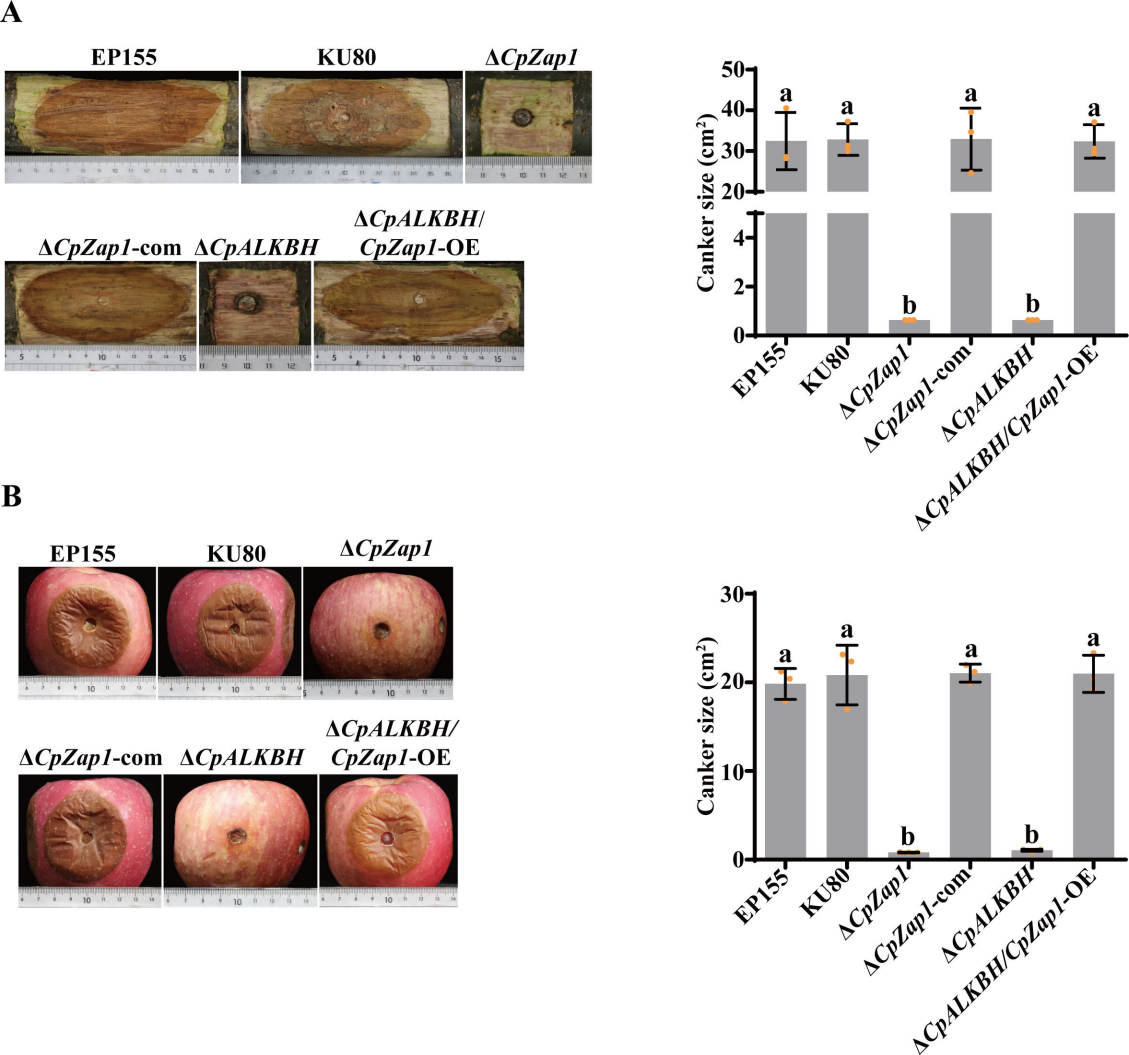
Virulence assay of *CpZap1* mutant strains. (**A**) The dormant Chinese chestnut stems were inoculated with the tested strains and kept at 26°C for 25 days. (**B**) The Red Fuji apples were inoculated with the tested strains at 26°C for 10  days. A standard deviation from three independent experiments is shown with the error bars. Treatment differences are indicated by different letters above the bars (ANOVA followed by Tukey’s test, *P* < 0.05).

To investigate the function of *CpZap1* in the development and virulence of *C. parasitica*, a *CpZap1* null mutant was generated by replacing *CpZap1* with the *hph* gene. PCR and Southern blotting were used to screen and confirm the single-spored transformants. Additionally, wild-type *CpZap1* was introduced into the ∆*CpZap1* to complement the mutant (Fig. 7A and B). In addition, we introduced *CpZap1* into the Δ*CpALKBH* mutant, which was named ∆*CpALKBH/CpZap1*-OE. qRT-PCR analysis revealed that the expression level of *CpZap1* was significantly increased in this mutant compared with ∆*CpALKBH* (Fig. S5). The growth rate and sporulation of ∆*CpZap1* significantly reduced compared to those of the wild-type strains (Fig. 7C and D). The observed abnormal phenotypes were successfully restored in the complemented strain ∆*CpZap1*-com. The ∆*CpALKBH/CpZap1*-OE strain showed a phenotype similar with that of the wild-type strain. Moreover, the virulence tests on chestnut stems and red Fuji apples were performed. The findings showed that the pathogenicity of ∆*CpZap1* mutants was completely lost, and the virulence of ∆*CpZap1*-com strains could be restored to the wild-type level. Moreover, ∆*CpALKBH/CpZap1*-OE strains incited cankers similar with those of the EP155 and KU80 strains (Fig. 8A and B). This indicates that *CpZap1* plays an important role in *C. parasitica* development and virulence.

To elucidate the crucial role of the m^6^A methylation site of *CpZap1* in *C. parasitica*, we introduced the extended *CpZap1* (CDS + 3′UTR) with key residue 1935A into the Δ*CpALKBH* mutant, named Δ*CpALKBH*/CpZap1 + 3′UTR. Furthermore, we mutated the m^6^A methylation site of *CpZap1* by changing A to C, resulting in the transformant Δ*CpALKBH*/CpZap1 + 3′UTR (A1935C) (Fig. S6A). Compared with the KU80 and Δ*CpALKBH* strains, the Δ*CpALKBH*/CpZap1 + 3′UTR strain grew much more slowly and showed significantly decreased conidial spores and virulence. However, the phenotype and virulence of the site-specific mutant Δ*CpALKBH*/CpZap1 + 3′UTR (A1935C) were similar to the KU80 strain (Fig. S6B and C). Furthermore, the m^6^A level of *CpZap1* in the Δ*CpALKBH*/CpZap1 + 3′UTR was significantly elevated compared to that in KU80 and Δ*CpALKBH*. In contrast, theA1935C mutation resulted in a substantial decrease in the m^6^A level of *CpZap1* (Fig. S6D). Additionally, the Δ*CpALKBH*/CpZap1 + 3′UTR strain demonstrated a marked decrease in the stability of *CpZap1* mRNA, while the mutation of A1935C did not (Fig. S6E). This finding suggests that the methylation of 1935A plays an important role in regulating the *CpZap1* mRNA stability and influencing the observed phenotypic switch in *C. parasitica*.

To further explore the regulatory mechanism of *CpZap1*, RNA-seq was conducted on the KU80 and ∆*CpZap1*. This analysis identified 1,056 upregulated genes and 1,693 downregulated genes in the ∆*CpZap1* strain compared to the KU80 strain (Fig. S7A; Table S4). The reliability of the RNA-seq data was verified by performing qRT-PCR analysis of eight randomly selected DEGs. A strong correlation (correlation coefficient *R*^2^ = 0.8424, *P* < 0.001) between qRT-PCR and RNA-seq results further validated their consistency (Fig. S7B; Table S4). To elucidate the primary functional categories represented by the DEGs, GO enrichment analysis was performed (Fig. S8A). The upregulated genes were mostly involved in ion transport, metal ion transport, intrinsic component of membrane, ion transmembrane transporter activity, and active transmembrane transporter activity. The downregulated DEGs were significantly enriched in mitotic DNA replication, nuclear DNA replication, nuclear replication fork progression, and catalytic activity. Moreover, we performed an enrichment analysis based on the KEGG pathways (Fig. S8B). The most enriched pathways for the upregulated genes were amino sugar and nucleotide sugar metabolism, MAPK signaling pathway, and tryptophan metabolism. However, the downregulated genes were enriched in DNA replication, mismatch repair, nucleotide excision repair, and cell cycle.

### Overlapping genes between Δ*CpALKBH* and Δ*CpZap1*

**Fig 9:**
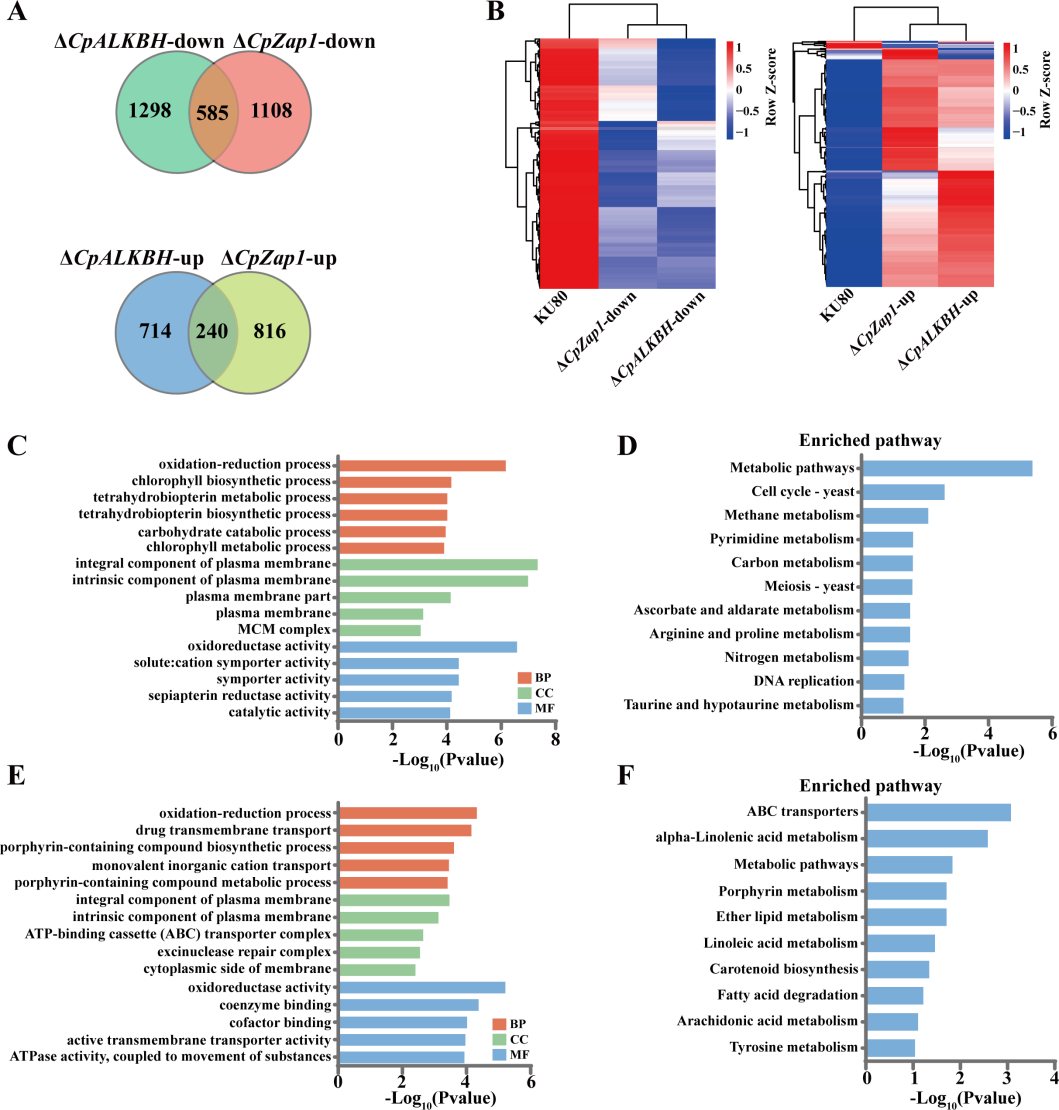
Analysis of overlapping genes between Δ*CpALKBH* and Δ*CpZap1* based on RNA-seq results. (**A**) Venn diagrams displaying the overlapped DEGs from RNA-seq (downregulated in Δ*CpALKBH* and Δ*CpZap1*, upregulated in Δ*CpALKBH* and Δ*CpZap1*). (**B**) The heat map was generated based on the expression levels shown in (**A**). (**C**) GO enrichment analysis of overlapping genes that were downregulated in both Δ*CpALKBH* and Δ*CpZap1*. (**D**) KEGG pathway enrichment analysis of overlapping genes that were downregulated in both Δ*CpALKBH* and Δ*CpZap1*. (**E**) GO enrichment analysis of overlapping genes that were upregulated in both Δ*CpALKBH* and Δ*CpZap1*. (**F**) KEGG pathway enrichment analysis of overlapping genes that were upregulated in both Δ*CpALKBH* and Δ*CpZap1*.

As both Δ*CpALKBH* and Δ*CpZap1* strains exhibited similar virulence deficiency, we reasoned that *CpALKBH* and *CpZap1* might function through a similar molecular mechanism. To address this, DEGs induced by *CpALKBH* and *CpZap1* deletion were chosen for further analysis. The overlapping analysis showed that 585 genes were downregulated in both Δ*CpALKBH* and Δ*CpZap1* strains. Similarly, 240 genes were upregulated in both Δ*CpALKBH* and Δ*CpZap1* strains (Fig. 9A and B; Table S5). The high number of overlapping DEGs between Δ*CpALKBH* and Δ*CpZap1* indicates that they function similarly. To demonstrate the biological function of these overlapping DEGs, we performed GO and KEGG analyses. GO analysis revealed significantly enriched functional terms for 585 overlapping downregulated genes, including oxidation-reduction process, plasma membrane, and oxidoreductase activity (Fig. 9C). In KEGG analysis, significantly enriched pathways, including metabolic pathways, cell cycle, meiosis, and DNA replication, were found (Fig. 9D). Additionally, GO analysis showed significant enrichment in drug transmembrane transport, ATP-binding cassette (ABC) transporter complex, coenzyme binding, and ATPase activity for these 240 overlapping upregulated genes (Fig. 9E). In KEGG analysis, significantly enriched pathways, including ABC transporters and metabolic pathways, were found (Fig. 9F).

## DISCUSSION

**Fig 10:**
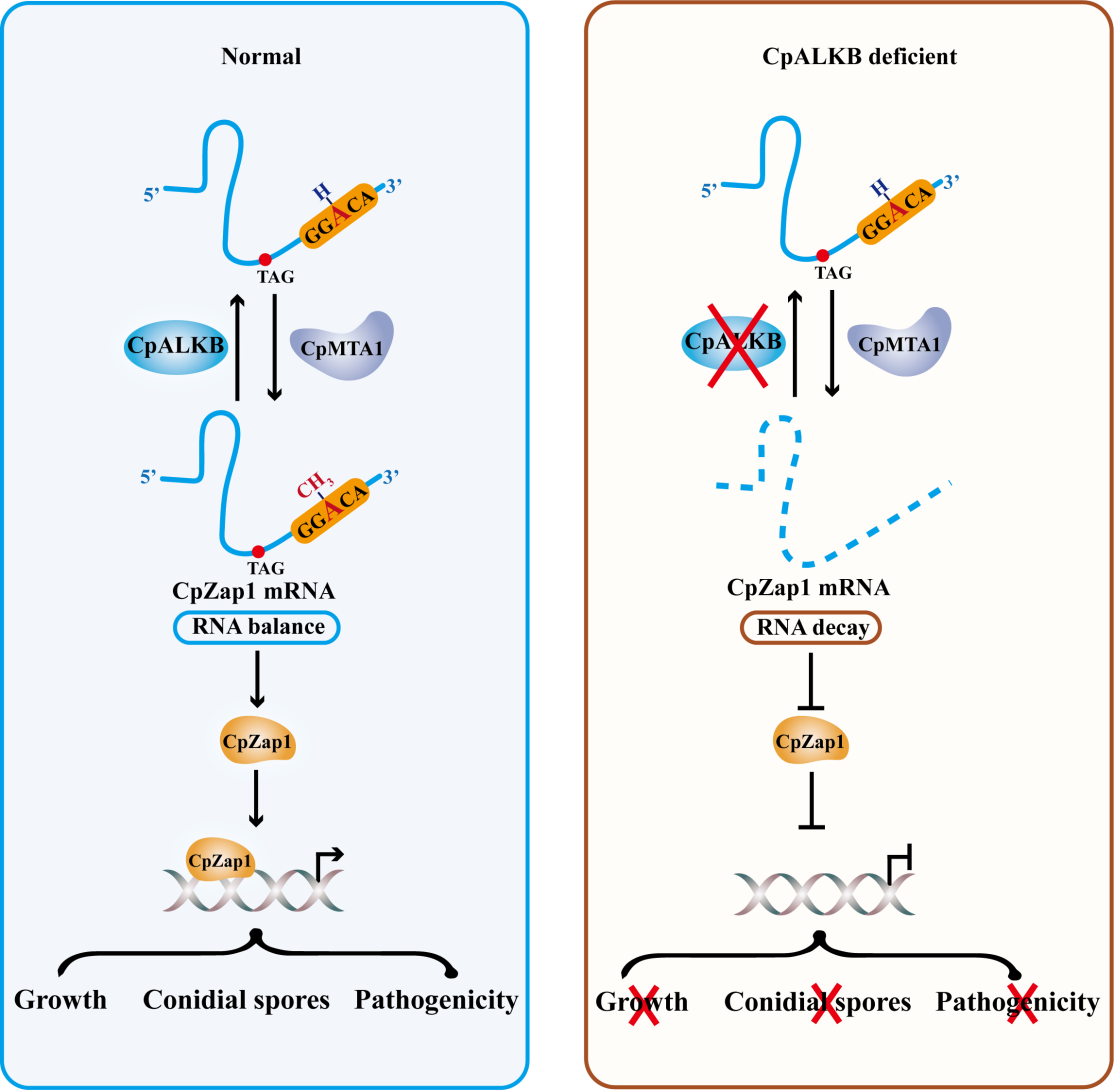
A proposed model for CpALKBH regulates the mRNA stability of transcription factor CpZap1 via an m^6^A-dependent manner. In wild-type strains, CpMTA1 catalyzes the m^6^A modification of *CpZap1*, while CpALKBH reverses this methylation. The m^6^A modification in *CpZap1* mRNA is balanced by both CpMTA1 and CpALKBH. However, in *CpALKBH* deletion mutants, the m^6^A level of *CpZap1* mRNA increased significantly compared with the control, which resulted in a reduction in the mRNA stability of *CpZap1*. As an important transcription factor, the decreased mRNA of *CpZap1* hinders the fungal growth, conidial spores, and pathogenicity of *C. parasitica*.

Recently, m^6^A modification has been identified as a pivotal mechanism in the regulation of mRNA biology (40). As a dynamic regulation process, methyltransferases and demethylases work together to regulate the m^6^A levels (41). Several studies have demonstrated that m^6^A modification is a conserved feature of mRNA and plays a critical regulatory role in fungi (23, 25, 26, 42). However, the biological effects of these m^6^A-modifying enzymes in phytopathogenic fungi remain largely unexplored. In this study, we report that CpALKBH can remove m^6^A modification from ssRNA *in vitro*, indicating that CpALKBH possesses m^6^A demethylase activity. Furthermore, we found that CpALKBH is essential for phenotypic characteristics and virulence of *C. parasitica*. In addition, CpALKBH regulates *CpZap1* mRNA stability in an m^6^A-dependent manner. *CpZap1* undergoes methylation at the 1935A position—a process regulated by the demethylase CpALKBH and methyltransferase CpMTA1. Moreover, *CpZap1* is involved in regulating the fungal phenotype and virulence. Figure 10 presents a schematic model that summarizes our findings.

The AlkB (ALKBH1-8, FTO) enzyme is a member of Fe^2+^- and α-KG-dependent dioxygenases, which catalyzes the demethylation of various substrates, including DNA, RNA, and histones. In mammals, ALKBH5 has been widely recognized as an m^6^A-specific demethylase (43). However, m^6^A demethylases have still not been identified in fungi. In a recent study reported by Shi et al. (25), a putative oxidoreductase, PoAlkb1, containing an ALKB domain was identified in *P. oryzae*. Although *PoAlkb1* was shown to be involved in virulence, it was not reported to be a demethylase. In this study, after comparing the *C. parasitica* genome with the human ALKBH5 sequence, an uncharacterized protein, XP_040772748 (CpALKBH)—a sole protein containing the Fe2OG dioxygenase domain in *C. parasitica—*was found. Subsequently, the CpALKBH protein was expressed in *E. coli* and its m^6^A demethylase activity was confirmed by RNA dot assay (Fig. 1). Similarly, ALKBH9B has been observed in *Arabidopsis* (21). Furthermore, we confirmed that *CpALKBH* deletion resulted in a notable increase of m^6^A levels, suggesting an important role of CpALKBH in the m^6^A modification in *C. parasitica* (Fig. 2). Consistent with our results, male mice deficient in *ALKBH5* also exhibited elevated levels of m^6^A in their mRNA (9).

As reported in previous studies, m^6^A methyltransferase deletion can impact various biological processes, including mouse fertility, antiviral response, animal and plant development, and tumor development (44–46). In this study, the Δ*CpALKBH* mutant exhibited a marked reduction in growth rate, sporulation, and virulence (Fig. 3), suggesting an essential role of *CpALKBH* in the regulation of *C. parasitica* development and virulence. Furthermore, we found that the impaired phenotype of *CpALKBH* deletion mutant might be related to the expression of metabolism-related genes (Fig. 4). The correlation analysis of this transcriptome with m^6^A distribution is an effective method to identify the downstream targets of m^6^A demethylase (35). Through overlapping analysis of the RNA‐seq data and previously obtained m^6^A-seq findings of the KU80 strain, a total of 184 upregulated genes and 423 downregulated genes with m^6^A modification have been identified as potential targets of CpALKBH. Among these genes, *CpZap1* was found to be crucial for CpALKBH functioning in *C. parasitica*. Although our focus was on the m^6^A modification of *CpZap1*, other target genes of CpALKBH may also affect CpALKBH functioning.

It has been extensively studied how m^6^A demethylase affects mRNA stability. For example, FTO specifically removes m^6^A methylation of mRNAs of DNA repair genes, leading to increased mRNA stability (47). In contrast, overexpression of ALKBH5 has been reported to significantly decrease PKMYT1 mRNA stability (48). The difference may lie in the position of m^6^A modification on mRNA. For instance, m^6^A within the 3′UTR or near the stop codon has been shown to reduce the stability of mRNA in a regular maize seedling, strawberry fruit, and tomato fruit. Conversely, m^6^A enrichment in the CDS region has been found to positively regulate the mRNA stability in the ripe strawberry fruit (49). The present study shows that the 1935A position of *CpZap1* is a key methylation modification site, which is located in the 3′UTR region (Fig. 5). Coincidently, the mRNA stability of *CpZap1* decreased in the Δ*CpALKBH* mutant, suggesting that the m^6^A methylation of *CpZap1* negatively mediates mRNA abundance. Nevertheless, further research is required to understand how m^6^A modification alters mRNA stability in *C. parasitica*.

As a transcription factor, CpZap1 belongs to the zinc finger protein family. In light of the highly conserved sequence of CpZap1 in eukaryotes, including fungi, plants, and animals, it is reasonable to speculate that the function of CpZap1 is crucial. In *S. cerevisiae*, Zap1 controls zinc metabolism by regulating the expression of zinc metabolism-related genes (50). The zinc sensors bZIP19 and bZIP23 in *Arabidopsis* maintain the zinc levels in plants (51). This study also revealed that *CpZap1* is necessary for *C. parasitica* development and virulence. Furthermore, the RNA-seq analysis confirmed that *CpZap1* influences the expression of a large number of genes, which are mostly enriched in amino sugar and nucleotide sugar metabolism, MAPK signaling pathway, DNA replication, mismatch repair, and nucleotide excision repair. This study represents the first report on the interaction between m^6^A modification and transcription factors in fungi, highlighting their significant roles in regulating gene expression and RNA metabolism. Such regulation is crucial for various cellular biological functions and developmental processes. Identifying whether m^6^A modification regulates the mRNA stability of zinc finger protein in other species would be of interest.

Until now, this study is the first to demonstrate that *CpZap1* is a direct downstream target of CpALKBH-regulated m^6^A demethylation in *C. parasitica*, revealing how CpALKBH influences *CpZap1* and modulates fungal phenotype and virulence. Collectively, these findings offer valuable insights into the role and mechanism of CpALKBH-mediated m^6^A demethylation in filamentous fungi, then enhancing the understanding of related studies.

## MATERIALS AND METHODS

The Materials and Methods within this manuscript have been previously described in our publications (39). They have been reproduced here for the convenience of the reader.

### Fungal strains and culture conditions

The wild-type strain EP155 of *C. parasitica* (ATCC 38755), its hypovirulent isogenic strain EP155/CHV1-EP713 (EP155 infected with hypovirus CHV1-EP713, ATCC 52571), the highly efficient gene deletion strain KU80 (∆*cpku80* of EP155) (52), and all the constructed mutants were maintained on potato dextrose agar (PDA) plates under a 12 h light/dark cycle at 26°C. These cultures were used for phenotypic analyses as well as for DNA and RNA preparation. The liquid complete medium was used to extract fungal proteins as described earlier (53).

### Purification of CpALKBH protein

The coding sequence of CpALKBH was amplified through polymerase chain reaction (PCR) from EP155 and then inserted into the pET32a expression vector. The resulting plasmid was named as pET32a-CpALKBH, and plasmid DNA was then transformed into *Escherichia coli* BL21. The expression of CpALKBH protein was induced with 0.5 mM isopropyl-beta-d-thiogalactopyranoside for 12 h at 16°C. The CpALKBH protein was purified from the supernatant through nickel affinity chromatography. Purified proteins were quantified using BCA (Pierce) assays and then analyzed by SDS-PAGE. The excess protein was stored at −80°C. Primer sequences are shown in Table S6.

### Three-dimensional structure prediction of CpALKBH protein

The three-dimensional structure of the CpALKBH protein was predicted utilizing AlphaFold2. Initially, the amino acid sequence of the CpALKBH protein was obtained from the NCBI database and stored in FASTA format. Subsequently, the AlphaFold2 (https://github.com/deepmind/alphafoldRoseTTAFold) prediction script was executed via the command line, with the input FASTA file and output directory specified. Upon completion, the predicted model was saved in PDB format and subjected to visualization and analysis using PyMOL software (https://pymol.org/2/). To identify the DSBH (Double-Strand Beta-Helix) domain, the structural comparison was conducted using PyMOL between the CpALKBH protein and the human ALKBH5 protein (PDB DOI: https://doi.org/10.2210/pdb4NJ4/pdb).

### m^6^A demethylase activity assay

RNA dot blot assay was performed to detect the m^6^A demethylase activity of CpALKBH as described earlier (21). Briefly, 2.5 µg of CpALKBH protein was incubated with m^6^A monomethylated ssRNA (Sangon Biotech) oligonucleotide at 25°C for 3 h in a reaction mixture including 50 mM phosphate buffer saline (pH 7.3), 10 µM α-ketoglutarate, 100 µM l-ascorbic acid ascorbate, and 20 µM (NH_4_)_2_Fe(SO_4_)_2_·6H_2_O. The heat-inactivated CpALKBH protein lacking any enzymatic activity was used as a control. The reaction was quenched by heating to 95°C for 10 min. RNA was extracted and spotted onto the Hybond-N+membrane. After drying, the membrane was UV crosslinked for 3 min at 1500 mJ/ cm^2^. The membrane was blocked with 5% non-fat milk in tris buffered saline tween (TBST) for 1 h before incubating with the m^6^A antibody (EpiGentek) overnight at 4°C. Ethylene blue staining was conducted using a 0.02% methylene blue solution in sodium acetate, serving as a loading control. Following TBST washing, a secondary antibody was incubated at room temperature for 1 h. Visualization was performed with the ECL detection system (AI600 images).

### Mutant construction and verification

The *CpALKBH* deletion mutant was constructed through homologous recombination (52). The upstream and downstream sequences of *CpALKBH* and the hygromycin B resistance gene were amplified using EP155 genomic DNA and pCPXHY2 plasmid (31), respectively. Through overlapping PCR, these three fragments were connected, and the fused cassette was then transformed into KU80 protoplast. The PDA supplemented with hygromycin was used to select the resistant transformants. The positive single-spored Δ*CpALKBH* deletion mutants were verified using PCR, reverse transcription PCR (RT-PCR), and Southern blotting. Subsequently, single-spore isolation was performed to purify these mutants (54). To construct the complementation strain, Δ*CpALKBH*-com, the open reading frame (ORF) and promoter of CpALKBH, were amplified and cloned into the vector pCPXG418 (55), which includes the resistance G418 marker. After validation through DNA sequencing, the vector pCPXG418-CpALKBH was introduced into the ∆*CpALKBH* deletion strain protoplast. The transformants that exhibited resistance to both G418 and hygromycin were selected, and subjected to PCR screening of the complementation construct, which was subsequently validated through Southern blotting.

To generate strains that express a fusion protein of CpALKBH with a 3 × Flag tag at the N-terminus, the *CpALKBH* ORF was inserted into a modified pCPXG418-3 × Flag vector. The pCPXG418-3 × Flag-CpALKBH plasmid was introduced into the EP155 strain. PCR and Western blot analysis were used to confirm the expression of CpALKBH. The gene deletion, complementation, and overexpression constructs of *CpZap1* were generated by similar methods as described above. Table S6 lists all primer sequences used for constructing and verifying fungal mutants.

### Fungal phenotype and virulence analysis

As a comparison to EP155, KU80, and EP155/CHV1-EP713, the phenotypic characteristics and virulence of the constructed mutants were analyzed. Phenotypic alterations in pigmentation, growth rate, and conidiation were assessed (56). After 14 days of culturing fungi on PDA, conidia were eluted with sterile water and counted using a hemacytometer. Fungal virulence was measured with the strain samples by inoculating the dormant Chinese chestnut (*Castanea mollissima*) stems and red Fuji apple. Incubation of the stems and apples at 26°C for 25 and 10 days, respectively. The canker size was observed and quantified according to Shi et al (57).

### RNA extraction and quantitative real-time PCR

The MiniBEST plant RNA extraction kit (Takara) was used to extract total RNA from *C. parasitica*, and the HiScript III RT supermix (Vazyme Biotech, R323) was used to prepare cDNA. Using SYBR green PCR master mix (Takara), qRT-PCR was carried out on the LightCycler480II real-time PCR system (Roche). Following the completion of the reactions, relative gene expression levels were quantified based on the 2^−ΔΔCt^ method. 18S rRNA served as a reference gene (58). A list of the primers used for qRT-PCR can be found in Table S6.

### Measurement of m^6^A RNA methylation levels

The colorimetric EpiQuik m^6^A RNA methylation quantification kit (EpiGentek, USA) was used to quantify the m^6^A RNA methylation level based on the manufacturer’s instructions. The assay wells were coated with 200 ng RNA or the m^6^A standard and then capture and detection antibodies were added. m^6^A levels were determined by measuring the absorbance at 450 nm (OD_450_) in each well. Subsequently, the relative m^6^A RNA methylation levels were quantified.

### Methylated RNA immunoprecipitation coupled with qRT-PCR

The methylated RNA immunoprecipitation (MeRIP) assay was conducted according to the manufacturer’s instructions using the m^6^A RNA enrichment kit (EpiGentek, USA). A total of 10 µg of RNA was extracted from each sample and subsequently fragmented into segments of 300 nucleotides or fewer. RNA samples were immunoprecipitated using magnetic beads coated with 10 µg of anti-m^6^A antibody or IgG. The product of IP or input assay was subsequently subjected to analysis via qRT-PCR utilizing special primers (Table S6).

### RNA immunoprecipitation

The RNA immunoprecipitation (RIP) assay was performed using the RNA Immunoprecipitation (RIP) Kit (BerSinBio, Bes5101) according to the manufacturer’s instructions. In brief, fungal cells were collected and lysed by RIP lysis buffer on ice, and DNA contamination was removed by DNase treatment. Subsequently, the cell lysate was immunoprecipitated with anti-Flag antibodies (Abmart) at 4℃ for 16 h, followed by a 1 h incubation with protein A/G beads. Finally, qRT-PCR was used to quantify the co-precipitated RNA. Table S6 lists the primers used in this assay.

### MazF RNA restriction enzyme assay

MazF (TaKaRa 2415A) was used to detect the methylation site on *CpZap1* mRNA (59). Initially, a total of 12.5 µg of RNA was used for the isolation of poly(A) mRNA, employing VAHTS mRNA capture beads (Vazyme) in accordance with the manufacturer’s protocol. In a total volume of 20 µL, the isolated mRNA was combined with MazF buffer (4  µL), RNase inhibitor (0.5  µL), and MazF enzyme (1  µL) solution. Subsequently, RNA samples were resuspended in RNase-free water, and cDNA synthesis was conducted using the HiScript III RT supermix kit (Vazyme Biotech, R323). The resulting cDNA was used as a template for PCR with primers (Table S6), and the products were analyzed on a 1% agarose TBE gel.

### Analysis of RNA-seq

Based on three biological replicates per strain, RNA-seq was performed on the *C. parasitica* strains KU80 and Δ*CpALKBH*. Total RNA was extracted; library was constructed and sequenced with Illumina HiSeqTM 4000 in Gene Denovo Biotechnology Co (Guangzhou, China). High-quality clean reads were obtained by using Fastp software (v0.18.0) to filter out reads with adapters, over 10% unknown nucleotides, and low-quality reads (Q-value ≤20) (60). With HISAT2 v2.4 software (61), clean data from each sample were mapped to the genome of *C. parasitica* (https://genome.jgi.doe.gov/portal/Crypa2/Crypa2.download.ftp.html). Differentially expressed genes (DEGs) were analyzed using DESeq2 (62). DEGs were considered significant if their log_2_(fold change) >1 and their false discovery rate <0.05.

### Western blot

Fungal proteins were extracted using NP40 lysis buffer (Solarbio) according to the previously described method (63). Subsequently, the protein was subjected to boiling with SDS loading buffer followed by separation with 12% SDS-PAGE and transfer onto a PVDF membrane (Millipore, USA). After the membrane was blocked, anti-Flag (Abmart) or anti-actin (ABclonal) antibodies were incubated overnight at 4°C. As a final step, ECL detection reagents (Coolaber, China) were used to identify the membrane.

### RNA decay assay

The stability of *CpZap1* mRNA in KU80 and Δ*CpALKBH* strains was detected using actinomycin D (GlpBio Technology, CA, USA). These strains were cultured in the EP liquid medium and then treated with 20 µM actinomycin D (resuspended in dimethyl sulfoxide). The strains inoculated in the same volume of dimethyl sulfoxide were used as controls. For RNA extraction, fungal samples were collected after 0, 8, and 24 h. qRT-PCR was used to measure the expression level of *CpZap1* mRNA (64).

### Statistical analysis

Analysis of variance (ANOVA) and student’s *t*-test were conducted using IBM SPSS Statistics 22 software. All data and error bars are presented as the mean ± SD from at least three independent experiments. Statistical significance was defined as a *P*-value less than 0.05. Unless otherwise noted, all experiments were conducted in triplicate.

## Data Availability

All data necessary for the evaluation of the conclusions presented in the paper are included within the main text and the Supporting Information. RNA-seq raw data were submitted to the sequence read archive database (SRA) under accession number SRP531574.

## SUPPLEMENTAL MATERIAL

Supplemental Figures mbio.01844-24-s0001.docx Figures S1 to S8.

Tables S1 to S4 mbio.01844-24-s0002.xlsx Raw data, and results for differentially expressed genes.

Table S5 mbio.01844-24-s0003.xlsx CpALKBH-up and CpZap1-up; CpALKBH-down and CpZap1-down.

Table S6 mbio.01844-24-s0004.xlsx Primers used in this study.

ASM does not own the copyrights to Supplemental Material that may be linked to, or accessed through, an article. The authors have granted ASM a non-exclusive, world-wide license to publish the Supplemental Material files. Please contact the corresponding author directly for reuse.

## References

[1] Liu WW, Zheng SQ, Li T, Fei YF, Wang C, Zhang S, Wang F, Jiang GM, Wang H. 2024. RNA modifications in cellular metabolism: implications for metabolism-targeted therapy and immunotherapy. Signal Transduct Target Ther 9:70. doi:10.1038/s41392-024-01777-5 38531882 PMC10966055

[2] Wei C, Gershowitz A, Moss B. 1975. N^6^, O^2^′-dimethyladenosine a novel methylated ribonucleoside next to the 5′ terminal of animal cell and virus mRNAs. Nature New Biol 257:251–253. doi:10.1038/257251a0 1161029

[3] Zhou H, Rauch S, Dai Q, Cui X, Zhang Z, Nachtergaele S, Sepich C, He C, Dickinson BC. 2019. Evolution of a reverse transcriptase to map N^1^-methyladenosine in human messenger RNA. Nat Methods 16:1281–1288. doi:10.1038/s41592-019-0550-4 31548705 PMC6884687

[4] Meyer KD, Saletore Y, Zumbo P, Elemento O, Mason CE, Jaffrey SR. 2012. Comprehensive analysis of mRNA methylation reveals enrichment in 3’ UTRs and near stop codons. Cell 149:1635–1646. doi:10.1016/j.cell.2012.05.003 22608085 PMC3383396

[5] Roundtree IA, Evans ME, Pan T, He C. 2017. Dynamic RNA modifications in gene expression regulation. Cell 169:1187–1200. doi:10.1016/j.cell.2017.05.045 28622506 PMC5657247

[6] Yang Y, Hsu PJ, Chen YS, Yang YG. 2018. Dynamic transcriptomic m^6^A decoration: writers, erasers, readers and functions in RNA metabolism. Cell Res 28:616–624. doi:10.1038/s41422-018-0040-8 29789545 PMC5993786

[7] Liu N, Dai Q, Zheng G, He C, Parisien M, Pan T. 2015. N^6^-methyladenosine-dependent RNA structural switches regulate RNA-protein interactions. Nature New Biol 518:560–564. doi:10.1038/nature14234 PMC4355918 25719671

[8] Wang X, Lu Z, Gomez A, Hon GC, Yue Y, Han D, Fu Y, Parisien M, Dai Q, Jia G, Ren B, Pan T, He C. 2014. N^6^-methyladenosine-dependent regulation of messenger RNA stability. Nature New Biol 505:117–120. doi:10.1038/nature12730 PMC3877715 24284625

[9] Zheng G, Dahl JA, Niu Y, Fedorcsak P, Huang C-M, Li CJ, Vågbø CB, Shi Y, Wang W-L, Song S-H, et al.. 2013. ALKBH5 is a mammalian RNA demethylase that impacts RNA metabolism and mouse fertility. Mol Cell 49:18–29. doi:10.1016/j.molcel.2012.10.015 23177736 PMC3646334

[10] Meyer KD, Patil DP, Zhou J, Zinoviev A, Skabkin MA, Elemento O, Pestova TV, Qian SB, Jaffrey SR. 2015. 5' UTR m^6^A promotes cap-independent translation. Cell 163:999–1010. doi:10.1016/j.cell.2015.10.012 26593424 PMC4695625

[11] Zhong S, Li H, Bodi Z, Button J, Vespa L, Herzog M, Fray RG. 2008. MTA is an Arabidopsis messenger RNA adenosine methylase and interacts with a homolog of a sex-specific splicing factor. Plant Cell 20:1278–1288. doi:10.1105/tpc.108.058883 18505803 PMC2438467

[12] Cao G, Li HB, Yin Z, Flavell RA. 2016. Recent advances in dynamic m^6^A RNA modification. Open Biol 6:160003. doi:10.1098/rsob.160003 27249342 PMC4852458

[13] Duan HC, Wang Y, Jia G. 2019. Dynamic and reversible RNA N^6^-methyladenosine methylation. Wiley Interdiscip Rev RNA 10:e1507. doi:10.1002/wrna.1507 30252201

[14] Li T, Hu PS, Zuo Z, Lin JF, Li X, Wu QN, Chen ZH, Zeng ZL, Wang F, Zheng J, Chen D, Li B, Kang TB, Xie D, Lin D, Ju HQ, Xu RH. 2019. METTL3 facilitates tumor progression via an m^6^A-IGF2BP2-dependent mechanism in *colorectal carcinoma*. Mol Cancer 18:112. doi:10.1186/s12943-019-1038-7 31230592 PMC6589893

[15] Wei J, Liu F, Lu Z, Fei Q, Ai Y, He PC, Shi H, Cui X, Su R, Klungland A, Jia G, Chen J, He C. 2018. Differential m^6^A, m^6^A_m_, and m^1^A demethylation mediated by FTO in the cell nucleus and cytoplasm. Mol Cell 71:973–985. doi:10.1016/j.molcel.2018.08.011 30197295 PMC6151148

[16] Liu J, Huang H, Zhang M, Qing G, Liu H. 2023. Intertwined regulation between RNA m^6^A modification and cancer metabolism. Cell Insight 2:100075. doi:10.1016/j.cellin.2022.100075 37192910 PMC10120304

[17] Li L, Chen J, Wang A, Yi K. 2024. ALKBH5 regulates ovarian cancer growth via demethylating long noncoding RNA PVT1 in ovarian cancer. J Cell Mol Med 28:e18066. doi:10.1111/jcmm.18066 38098223 PMC10826426

[18] Liu L, Gu M, Ma J, Wang Y, Li M, Wang H, Yin X, Li X. 2022. CircGPR137B/miR-4739/FTO feedback loop suppresses tumorigenesis and metastasis of hepatocellular carcinoma. Mol Cancer 21:149. doi:10.1186/s12943-022-01619-4 35858900 PMC9297645

[19] Li Z, Weng H, Su R, Weng X, Zuo Z, Li C, Huang H, Nachtergaele S, Dong L, Hu C, et al.. 2017. FTO plays an oncogenic role in acute myeloid leukemia as a N^6^-methyladenosine RNA demethylase.. Cancer Cell 31:127–141. doi:10.1016/j.ccell.2016.11.017 28017614 PMC5234852

[20] Liu Y, Song R, Lu Z, Zhao L, Zhan X, Li Y, Cao X. 2024. The RNA m^6^A demethylase ALKBH5 drives emergency granulopoiesis and neutrophil mobilization by upregulating G-CSFR expression. Cell Mol Immunol 21:6–18. doi:10.1038/s41423-023-01115-9 38114747 PMC10757716

[21] Martínez-Pérez M, Aparicio F, López-Gresa MP, Bellés JM, Sánchez-Navarro JA, Pallás V. 2017. Arabidopsis m^6^A demethylase activity modulates viral infection of a plant virus and the m^6^A abundance in its genomic RNAs. Proc Natl Acad Sci U S A 114:10755–10760. doi:10.1073/pnas.1703139114 28923956 PMC5635872

[22] Corbeski I, Vargas-Rosales PA, Bedi RK, Deng J, Coelho D, Braud E, Iannazzo L, Li Y, Huang D, Ethève-Quelquejeu M, Cui Q, Caflisch A. 2024. The catalytic mechanism of the RNA methyltransferase METTL3. Elife 12:RP92537. doi:10.7554/eLife.92537 38470714 PMC10932547

[23] Yadav PK, Rajasekharan R. 2017. The m^6^A methyltransferase Ime4 epitranscriptionally regulates triacylglycerol metabolism and vacuolar morphology in haploid yeast cells. J Biol Chem 292:13727–13744. doi:10.1074/jbc.M117.783761 28655762 PMC5566527

[24] Ren Z, Tang B, Xing J, Liu C, Cai X, Hendy A, Kamran M, Liu H, Zheng L, Huang J, Chen XL. 2022. MTA1-mediated RNA m^6^A modification regulates autophagy and is required for infection of the rice blast fungus. New Phytol 235:247–262. doi:10.1111/nph.18117 35338654

[25] Shi Y, Wang H, Wang J, Liu X, Lin F, Lu J. 2019. N^6^-methyladenosine RNA methylation is involved in virulence of the rice blast fungus Pyricularia oryzae (syn. Magnaporthe oryzae). FEMS Microbiol Lett 366. doi:10.1093/femsle/fny286 30535195

[26] Yang C, Wu D, Lin H, Ma D, Fu W, Yao Y, Pan X, Wang S, Zhuang Z. 2024. Role of RNA modifications, especially m^6^A, in aflatoxin biosynthesis of Aspergillus flavus J Agric Food Chem 72:726–741. doi:10.1021/acs.jafc.3c05926 38112282

[27] Eusebio-Cope A, Sun L, Tanaka T, Chiba S, Kasahara S, Suzuki N. 2015. The chestnut blight fungus for studies on virus/host and virus/virus interactions: from a natural to a model host. Virology (Auckl) 477:164–175. doi:10.1016/j.virol.2014.09.024 25454384

[28] Shang J, Wu X, Lan X, Fan Y, Dong H, Deng Y, Nuss DL, Chen B. 2008. Large-scale expressed sequence tag analysis for the chestnut blight fungus Cryphonectria parasitica. Fungal Genet Biol 45:319–327. doi:10.1016/j.fgb.2007.11.002 18166491

[29] Wang J, Shi L, He X, Lu L, Li X, Chen B. 2016. Comparative secretome analysis reveals perturbation of host secretion pathways by a hypovirus. Sci Rep 6:34308. doi:10.1038/srep34308 27698384 PMC5048421

[30] Chen Q, Li Y, Wang J, Li R, Chen B. 2018. cpubi4 is essential for development and virulence in chestnut blight fungus. Front Microbiol 9:1286. doi:10.3389/fmicb.2018.01286 29963030 PMC6013588

[31] Li R, Chen F, Li S, Yuan L, Zhao L, Tian S, Chen B. 2023. Comparative acetylomic analysis reveals differentially acetylated proteins regulating fungal metabolism in hypovirus-infected chestnut blight fungus. Mol Plant Pathol 24:1126–1138. doi:10.1111/mpp.13358 37278715 PMC10423328

[32] Li R, Zhou S, Li Y, Shen X, Wang Z, Chen B. 2018. Comparative methylome analysis reveals perturbation of host epigenome in chestnut blight fungus by a hypovirus. Front Microbiol 9:1026. doi:10.3389/fmicb.2018.01026 29875746 PMC5974932

[33] Crouch JA, Dawe A, Aerts A, Barry K, Churchill ACL, Grimwood J, Hillman BI, Milgroom MG, Pangilinan J, Smith M, Salamov A, Schmutz J, Yadav JS, Grigoriev IV, Nuss DL. 2020. Genome sequence of the chestnut blight fungus Cryphonectria parasitica EP155: a fundamental resource for an archetypical invasive plant pathogen. Phytopathol 110:1180–1188. doi:10.1094/PHYTO-12-19-0478-A 32207662

[34] Feng C, Liu Y, Wang G, Deng Z, Zhang Q, Wu W, Tong Y, Cheng C, Chen Z. 2014. Crystal structures of the human RNA demethylase Alkbh5 reveal basis for substrate recognition. J Biol Chem 289:11571–11583. doi:10.1074/jbc.M113.546168 24616105 PMC4002068

[35] Zhang S, Zhao BS, Zhou A, Lin K, Zheng S, Lu Z, Chen Y, Sulman EP, Xie K, Bögler O, Majumder S, He C, Huang S. 2017. m^6^A demethylase ALKBH5 maintains tumorigenicity of glioblastoma stem-like cells by sustaining FOXM1 expression and cell proliferation program. Cancer Cell 31:591–606. doi:10.1016/j.ccell.2017.02.013 28344040 PMC5427719

[36] John E, Singh KB, Oliver RP, Tan KC. 2021. Transcription factor control of virulence in phytopathogenic fungi. Mol Plant Pathol 22:858–881. doi:10.1111/mpp.13056 33973705 PMC8232033

[37] Zhang Y, Zhang J, Hoeflich KP, Ikura M, Qing G, Inouye M. 2003. MazF cleaves cellular mRNAs specifically at ACA to block protein synthesis in Escherichia coli. Mol Cell 12:913–923. doi:10.1016/S1097-2765(03)00402-7 14580342

[38] Wang X, He C. 2014. Dynamic RNA modifications in posttranscriptional regulation. Mol Cell 56:5–12. doi:10.1016/j.molcel.2014.09.001 25280100 PMC7129666

[39] Zhao L, Wei X, Chen F, Yuan L, Chen B, Li R. 2024. N^6^-methyladenosine RNA methyltransferase CpMTA1 mediates CpAphA mRNA stability through a YTHDF1-dependent m6A modification in the chestnut blight fungus. PLoS Pathog 20:e1012476. doi:10.1371/journal.ppat.1012476 39159278 PMC11361730

[40] Lan Q, Liu PY, Haase J, Bell JL, Hüttelmaier S, Liu T. 2019. The critical role of RNA m^6^A methylation in cancer. Cancer Res 79:1285–1292. doi:10.1158/0008-5472.CAN-18-2965 30894375

[41] Meyer KD, Jaffrey SR. 2017. Rethinking m^6^A readers, writers, and erasers. Annu Rev Cell Dev Biol 33:319–342. doi:10.1146/annurev-cellbio-100616-060758 28759256 PMC5963928

[42] Kim H, Hu J, Kang H, Kim W. 2024. Phylogenetic and functional analyses of N^6^-methyladenosine RNA methylation factors in the wheat scab fungus Fusarium graminearum. mSphere 9:e0055223. doi:10.1128/msphere.00552-23 38085094 PMC10826363

[43] Li Q, Zhu Q. 2023. The role of demethylase AlkB homologs in cancer. Front Oncol 13:1153463. doi:10.3389/fonc.2023.1153463 37007161 PMC10060643

[44] Hong J, Xu K, Lee JH. 2022. Biological roles of the RNA m^6^A modification and its implications in cancer. Exp Mol Med 54:1822–1832. doi:10.1038/s12276-022-00897-8 36446846 PMC9722703

[45] Feng L, Li M, Ma J, Wang W, Wang S, Mao Z, Zhang Y. 2024. ALKBH5 regulates arginase 1 expression in MDSCs and their immunosuppressive activity in tumor-bearing host. Noncoding RNA Res 9:913–920. doi:10.1016/j.ncrna.2024.03.003 38638146 PMC11024866

[46] Karandashov I, Kachanov A, Dukich M, Ponomareva N, Brezgin S, Lukashev A, Pokrovsky VS, Chulanov V, Kostyusheva A, Kostyushev D. 2024. m^6^A methylation in regulation of antiviral innate immunity. Viruses 16:601. doi:10.3390/v16040601 38675942 PMC11054785

[47] Zhang Q, Riddle RC, Yang Q, Rosen CR, Guttridge DC, Dirckx N, Faugere MC, Farber CR, Clemens TL. 2019. The RNA demethylase FTO is required for maintenance of bone mass and functions to protect osteoblasts from genotoxic damage. Proc Natl Acad Sci USA 116:17980–17989. doi:10.1073/pnas.1905489116 31434789 PMC6731662

[48] Hu Y, Gong C, Li Z, Liu J, Chen Y, Huang Y, Luo Q, Wang S, Hou Y, Yang S, Xiao Y. 2022. Demethylase ALKBH5 suppresses invasion of gastric cancer via PKMYT1 m^6^A modification. Mol Cancer 21:34. doi:10.1186/s12943-022-01522-y 35114989 PMC8812266

[49] Shen L, Ma J, Li P, Wu Y, Yu H. 2023. Recent advances in the plant epitranscriptome. Genome Biol 24:43. doi:10.1186/s13059-023-02872-6 36882788 PMC9990323

[50] Frey AG, Bird AJ, Evans-Galea MV, Blankman E, Winge DR, Eide DJ. 2011. Zinc-regulated DNA binding of the yeast Zap1 zinc-responsive activator. PLoS One 6:e22535. doi:10.1371/journal.pone.0022535 21799889 PMC3142189

[51] Lilay GH, Persson DP, Castro PH, Liao F, Alexander RD, Aarts MGM, Assunção AGL. 2021. Arabidopsis bZIP19 and bZIP23 act as zinc sensors to control plant zinc status. Nat Plants 7:137–143. doi:10.1038/s41477-021-00856-7 33594269

[52] Lan X, Yao Z, Zhou Y, Shang J, Lin H, Nuss DL, Chen B. 2008. Deletion of the cpku80 gene in the chestnut blight fungus, Cryphonectria parasitica, enhances gene disruption efficiency. Curr Genet 53:59–66. doi:10.1007/s00294-007-0162-x 17972079

[53] Puhalla JE. 1971. Genetics and nutritional requirements of Endothia parasitica. Phytopathology 61:169–173. doi:10.1094/Phyto-61-169

[54] Chong L. 2001. Molecular cloning - a laboratory manual. 3rd ed, Vol. 292, p 446. Science.

[55] Chen MM, Jiang M, Shang J, Lan X, Yang F, Huang J, Nuss DL, Chen B. 2011. CYP1, a hypovirus-regulated cyclophilin, is required for virulence in the chestnut blight fungus. Mol Plant Pathol 12:239–246. doi:10.1111/j.1364-3703.2010.00665.x 21355996 PMC3313458

[56] Kim D-H, Rigling D, Zhang L, NKVJMP-mI A. 1995. A new extracellular laccase of Cryphonectria parasitica is revealed by deletion of Lac1. MPMI 8:259–266. doi:10.1094/MPMI-8-0259

[57] Shi L, Li R, Liao S, Bai L, Lu Q, Chen B. 2014. Prb1, a subtilisin-like protease, is required for virulence and phenotypical traits in the chestnut blight fungus. FEMS Microbiol Lett 359:26–33. doi:10.1111/1574-6968.12547 25066598

[58] Livak KJ, Schmittgen TD. 2001. Analysis of relative gene expression data using real-time quantitative PCR and the 2^−ΔΔC_T_^ Method. Methods 25:402–408. doi:10.1006/meth.2001.1262 11846609

[59] Imanishi M, Tsuji S, Suda A, Futaki S. 2017. Detection of N^6^-methyladenosine based on the methyl-sensitivity of MazF RNA endonuclease. Chem Commun (Camb) 53:12930–12933. doi:10.1039/c7cc07699a 29154383

[60] Liu G, Wang J, Hou X. 2020. Transcriptome-wide N^6^-methyladenosine (m^6^A) methylome profiling of heat stress in Pak-choi (Brassica rapa ssp. chinensis). Plants (Basel) 9:1080. doi:10.3390/plants9091080 32842619 PMC7570095

[61] Kim D, Paggi JM, Park C, Bennett C, Salzberg SL. 2019. Graph-based genome alignment and genotyping with HISAT2 and HISAT-genotype. Nat Biotechnol 37:907–915. doi:10.1038/s41587-019-0201-4 31375807 PMC7605509

[62] Love MI, Huber W, Anders S. 2014. Moderated estimation of fold change and dispersion for RNA-seq data with DESeq2. Genome Biol 15:550. doi:10.1186/s13059-014-0550-8 25516281 PMC4302049

[63] Wang J, Lu L, Yang Y, Chen Q, Chen B. 2014. Proteomic analysis of Cryphonectria parasitica infected by a virulence-attenuating hypovirus. Wei Sheng Wu Xue Bao 54:803–812. 25252462

[64] Ratnadiwakara M, Änkö ML. 2018. mRNA stability assay using transcription inhibition by actinomycin D in mouse pluripotent stem cells. Bio Protoc 8:e3072. doi:10.21769/BioProtoc.3072 PMC8342049 34532533

