# Supplementary material for: m6A demethylase CpALKBH regulates CpZap1 mRNA stability to modulate the development and virulence of chestnut blight fungus
Source: mBio. 2024 Nov 29;16(1):e01844-24. doi: 10.1128/mbio.01844-24 (PMC11708048; doi:10.1128/mbio.01844-24)
Supplement: Supplemental Figures — Figures S1 to S8. [file mbio.01844-24-s0001.docx]

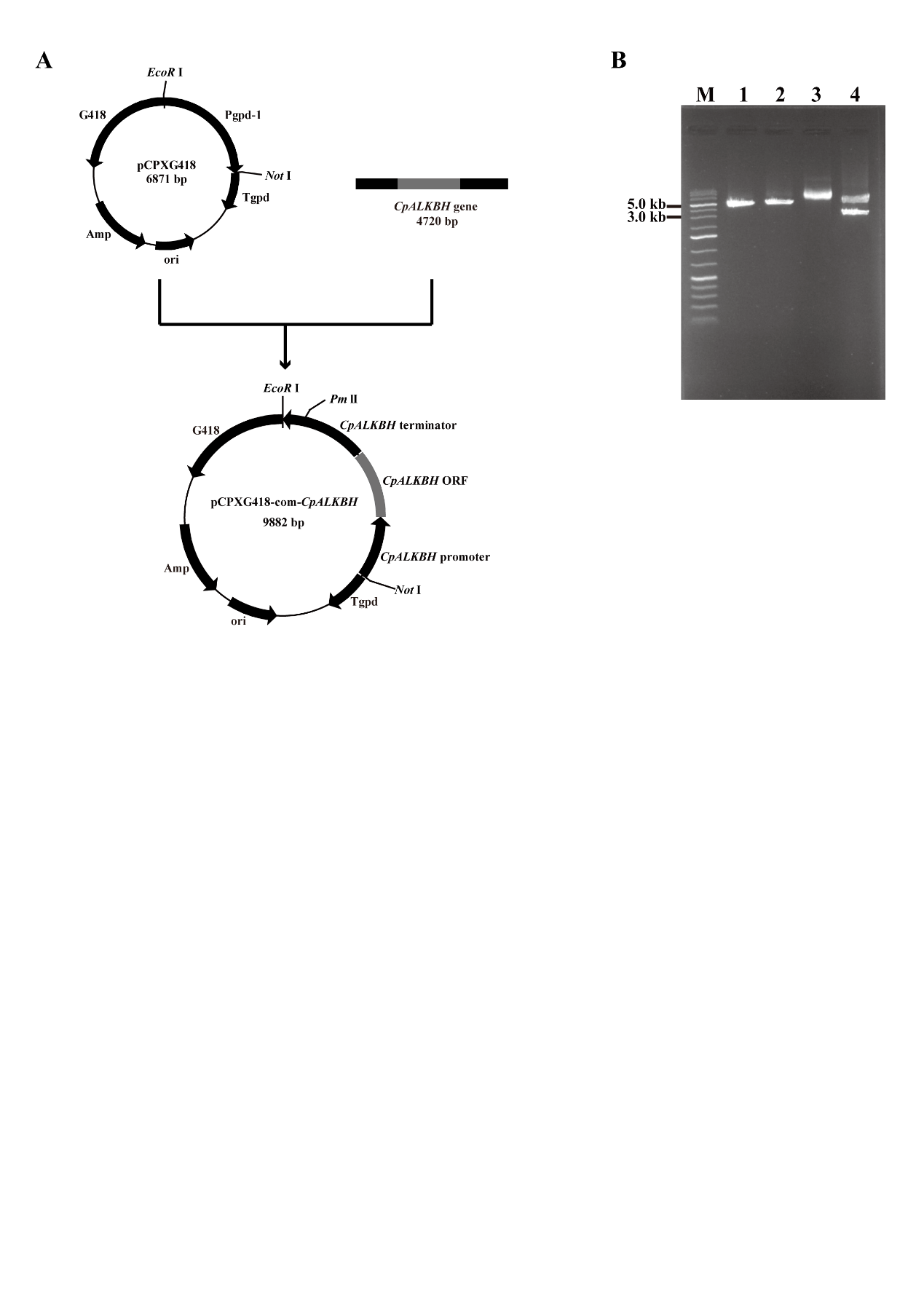


**Figure S1**

Construction and verification of plasmid pCPXG418-com-*CpALKBH*. A: Schematic diagram of the construction of *CpALKBH* gene complement plasmid pCPXG418-com-*CpALKBH*. B: Verification of the plasmid pCPXG418-com-*CpALKBH* using *Eco*R I/*Not* I digestion. Lane 1: The *CpALKBH* gene; lane 2: The pCPXG418 plasmid after *Eco*R I/*Not* I digestion; lane 3: The pCPXG418-com-*CpALKBH* plasmid; lane 4: The pCPXG418-com-*CpALKBH* plasmid after *Pm* II/Not I digestion.


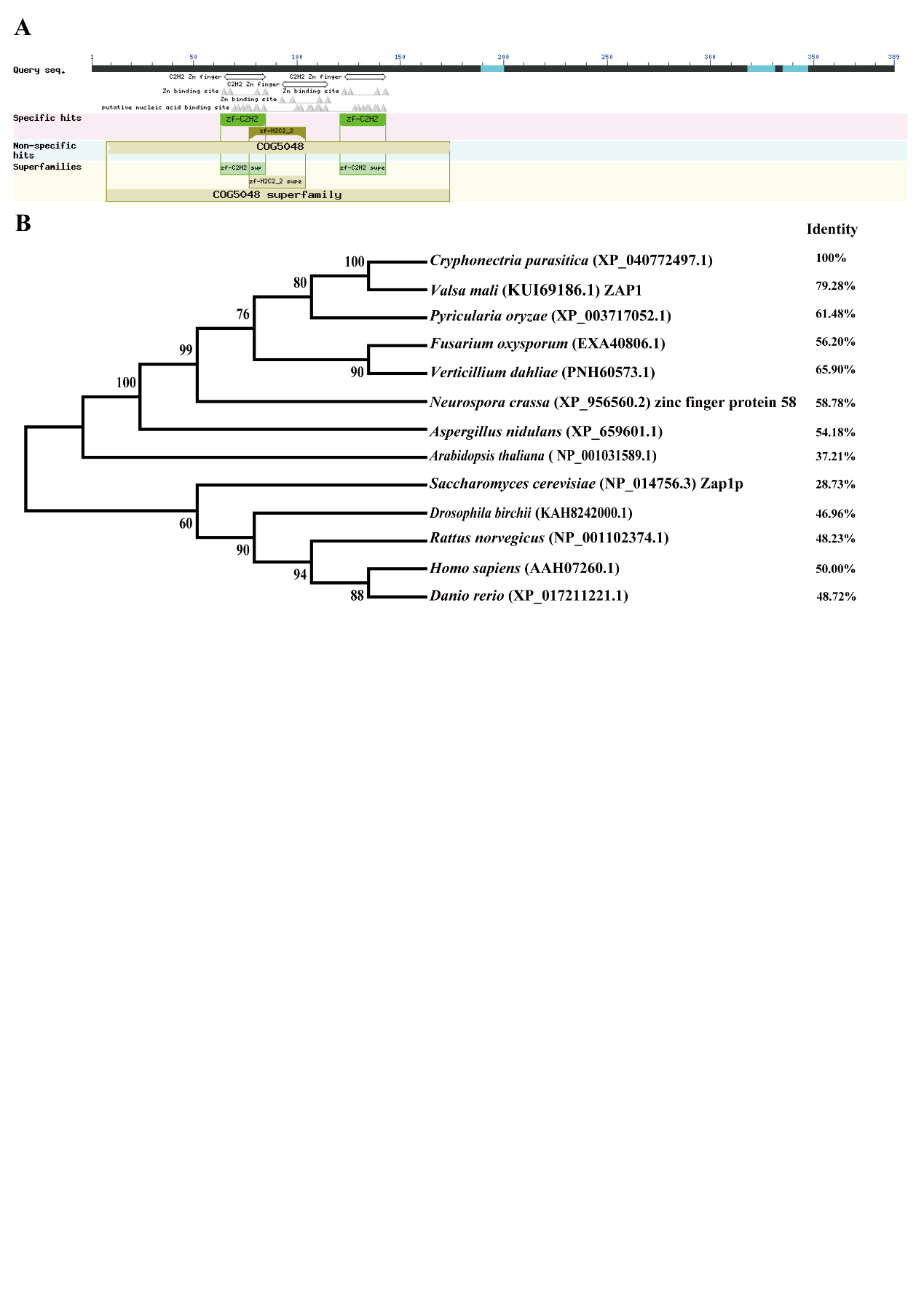


**Figure S2**

Sequence analysis of protein CpZap1. A: Analysis of conservative structural domains of CpZap1 in NCBI. B: Phylogenetic tree of CpZap1 orthologs from diverse species using MEGAX software analysis. The sequence similarity between CpZap1 and other homologous proteins was determined using DNAMAN software.


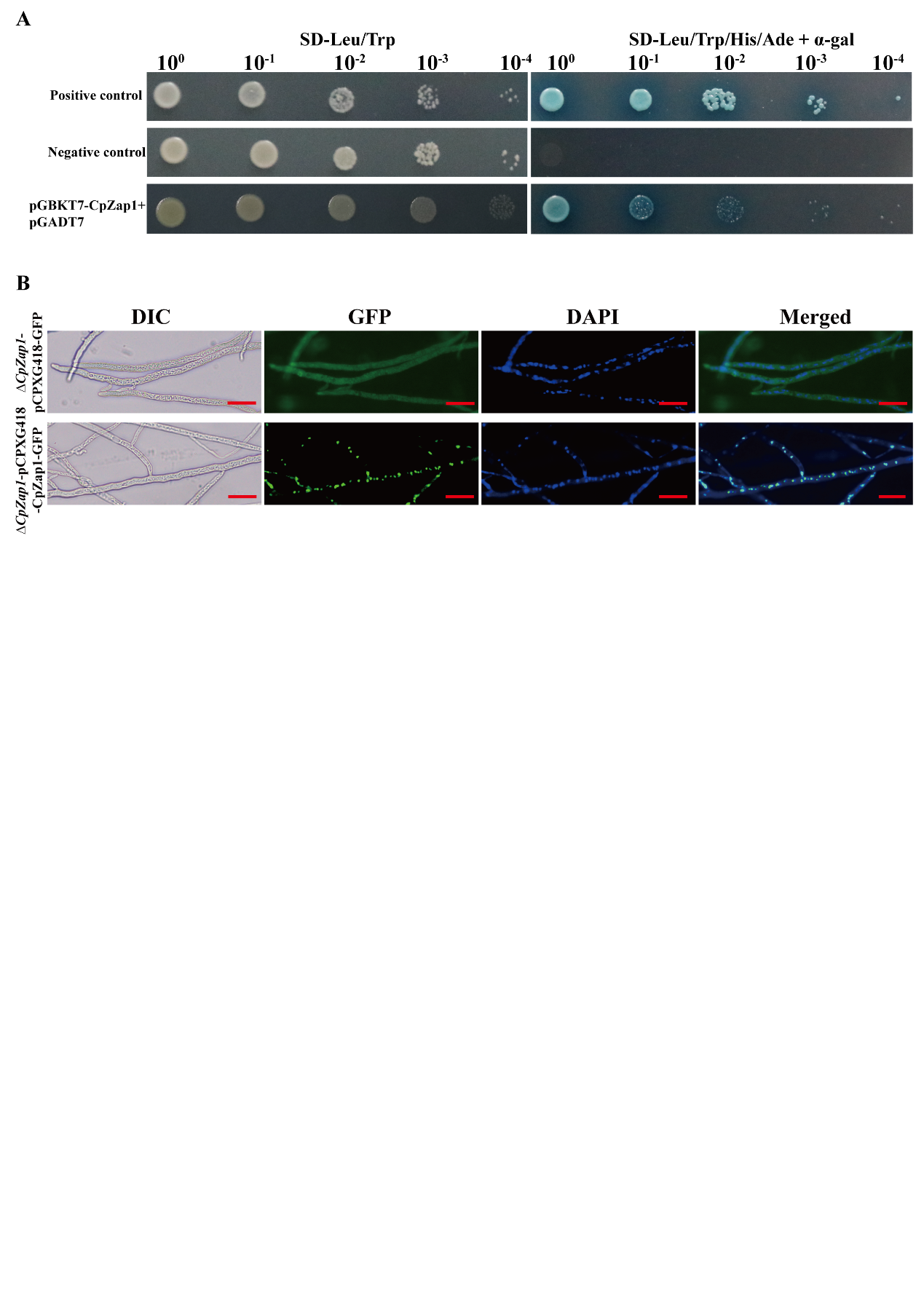


**Figure S3**

Transcriptional activation function and subcellular localization of CpZap1. A: Transcriptional activation function of CpZap1 was assessed by growing yeast cells containing different plasmids on SD-Leu/Trp medium (left) and on SD-Leu/Trp-His-Ade+α-gal medium (right). Positive control: pGBKT7-53+pGADT7-T. Negative control: pGBKT7-lam+ pGADT7-T. B: Subcellular localization of CpZap1-GFP fusion protein. Images were taken using light microscopy and fluorescence microscopy, respectively. DAPI staining was performed to visualize the nuclei. Scale bar, 20 μm.


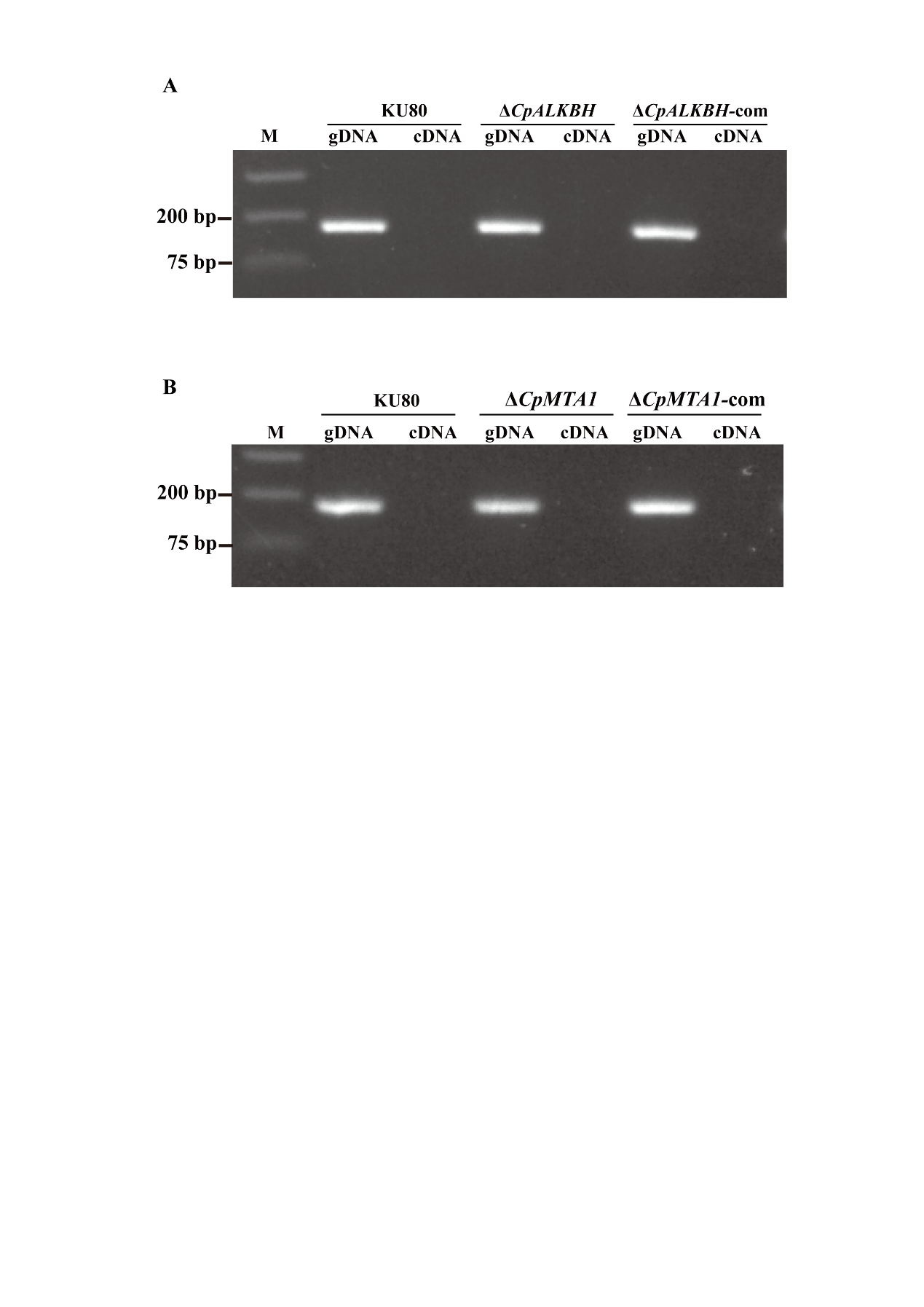


**Figure S4**

PCR analysis for *CpZap1* in gDNA and cDNA in KU80, Δ*CpALKBH* and Δ*CpALKBH*-com (A). PCR analysis for *CpZap1* in gDNA and cDNA in KU80, Δ*CpMTA1* and Δ*CpMTA1*-com (B).


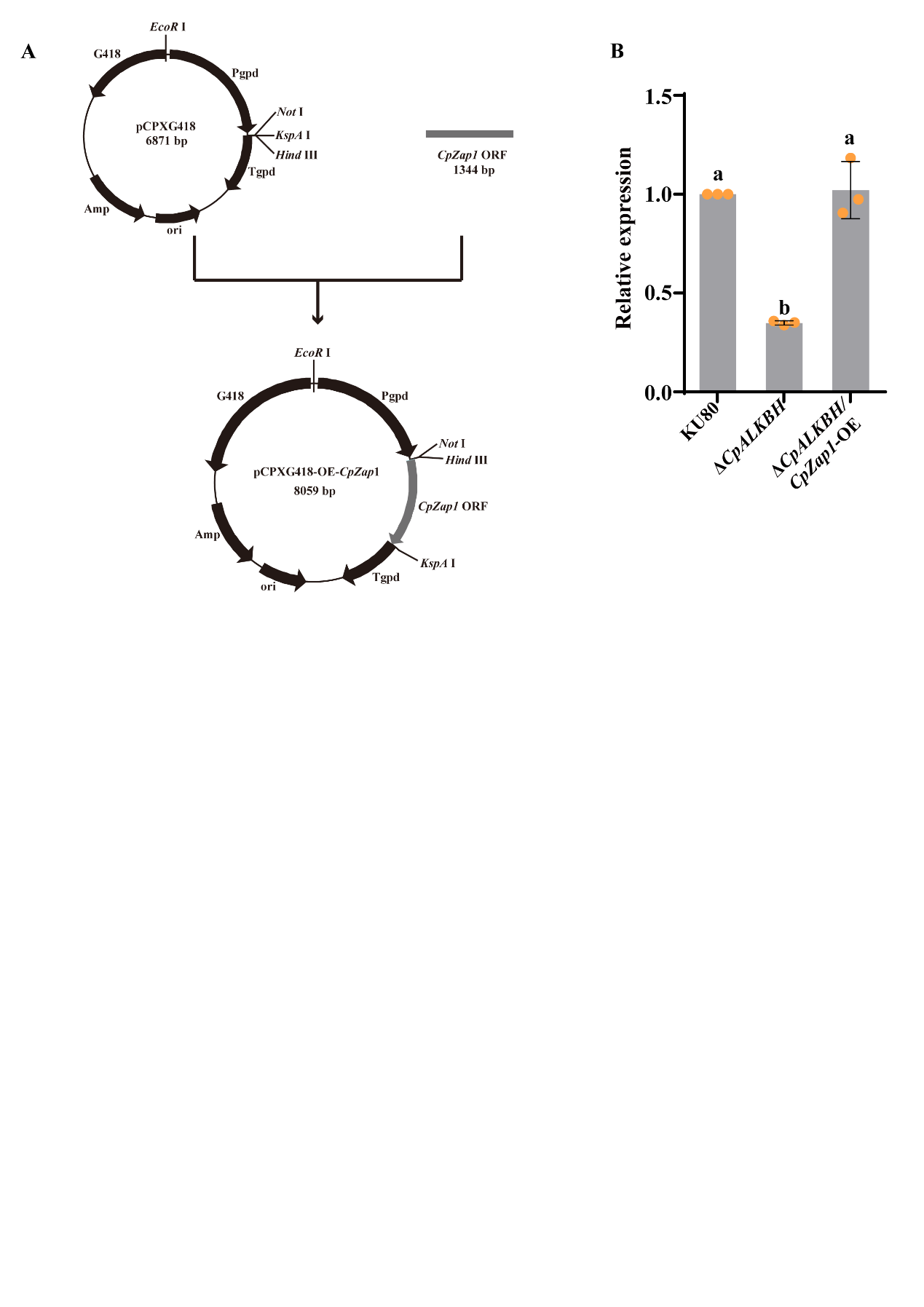


**Figure S5**

Construction of Δ*CpALKBH*/*CpZap1*-OE strain. A: Schematic diagram of the construction of *CpZap1* gene overexpression plasmid pCPXG418-OE-*CpZap1*. B: Analysis of *CpZap1* expression level using qRT-PCR. The expression level of *CpZap1*gene in KU80 was set as 1. The Error bars represent standard deviations from three independent experiments. Different letters above the bars indicate significant differences among treatments (ANOVA followed by Tukey’s test, p<0.05).


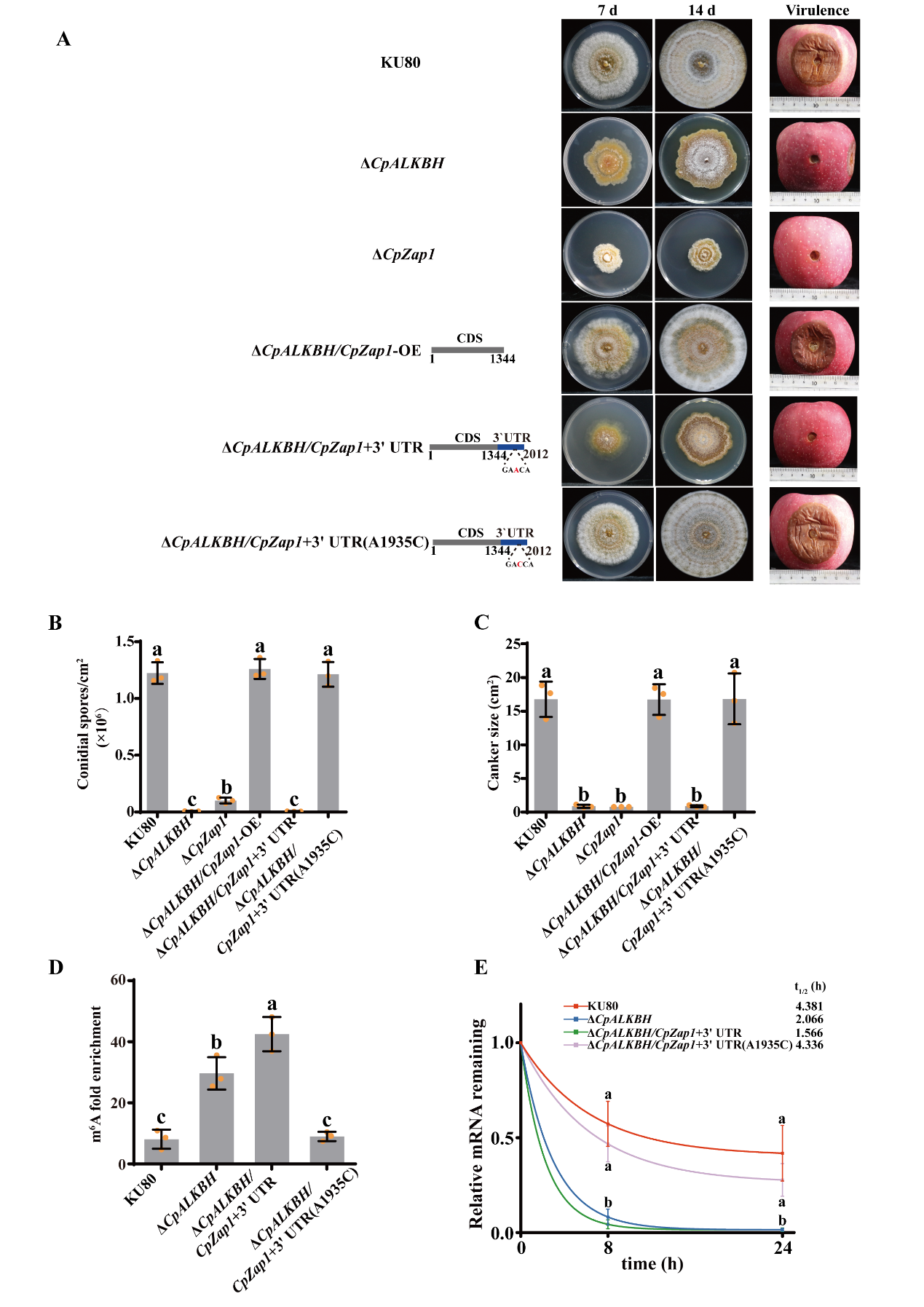


**Figure S6**

Analysis of phenotypes, sporulation and virulence in two different mutants (Δ*CpALKBH*/*CpZap1*+3’UTR and Δ*CpALKBH*/*CpZap1*+3’UTR (A1935C)). A: Colony morphology of the mutants on PDA plates. Photographs were taken at 7 and 14 days after inoculation. The strains shown were the parental strain KU80, the *CpALKBH*

deletion strain ∆*CpALKBH*, the complementary strain ∆*CpALKBH/CpZap1*-OE, Δ*CpALKBH*/*CpZap1*+3’UTR and Δ*CpALKBH*/*CpZap1*+3’UTR (A1935C). B: Sporulation levels of the indicated strains. Spores were analyzed on day 14. C: Cankers were induced by the tested strains on Red Fuji apples. The inoculated apples were maintained at 26°C, and cankers were assessed and photographed 10 days post-inoculation. D: MeRIP-qRT-PCR analysis with anti-m^6^A antibody was conducted to detect m^6^A levels of *CpZap1* in KU80, Δ*CpALKBH* mutant, Δ*CpALKBH*/*CpZap1*+3’UTR and Δ*CpALKBH*/*CpZap1*+3’UTR (A1935C). E: The *CpZap1* expression level was measured by qRT-PCR in KU80, Δ*CpALKBH* mutant, Δ*CpALKBH*/*CpZap1*+3’UTR and Δ*CpALKBH*/*CpZap1*+3’UTR (A1935C) after actinomycin D treatment. The mRNA half-life (t_1/2_) of *CpZap1* was assessed using a nonlinear regression model. Error bars represent standard deviations from three independent experiments. Different letters above the bars indicate significant differences among treatments (ANOVA followed by Tukey’s test, p<0.05).


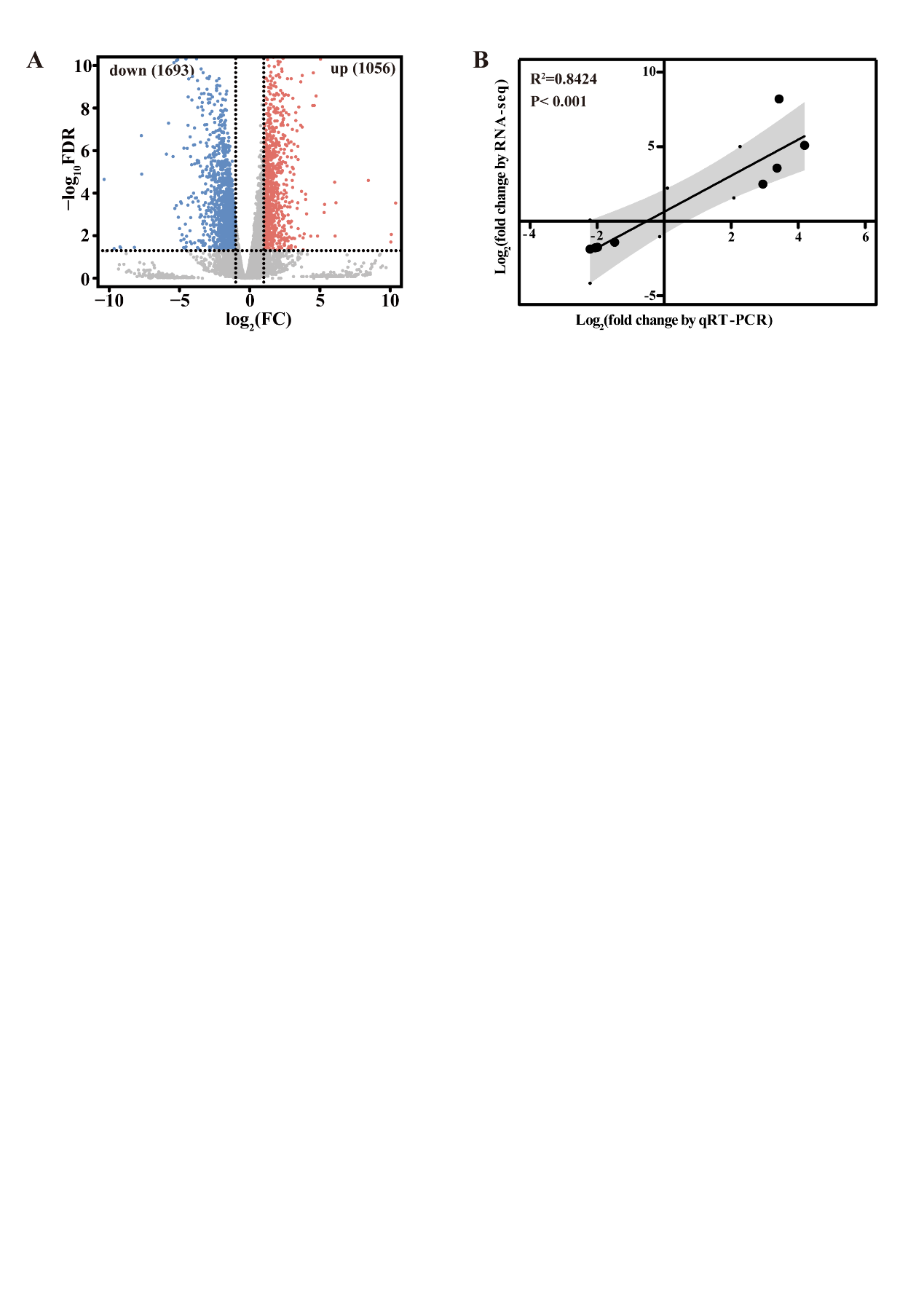


**Figure S7**

RNA-seq analysis identified transcripts affected by *CpZap1*. A: Volcano plot showing the DEGs between KU80 and Δ*CpZap1*. mRNAs log_2_FC >1 in Δ*CpZap1* relative to KU80 (p value <0.01, two-tailed t test) were highlighted in red. mRNAs log_2_FC < -1 in Δ*CpZap1* relative to KU80 (p value < 0.01, two-tailed t test) were highlighted in blue. B: Validation of the expression of 8 randomly selected DEGs using RNA-seq and qRT-PCR analysis. Log_2_FC was calculated from three samples. The coefficient of determination (R^2^) was displayed.


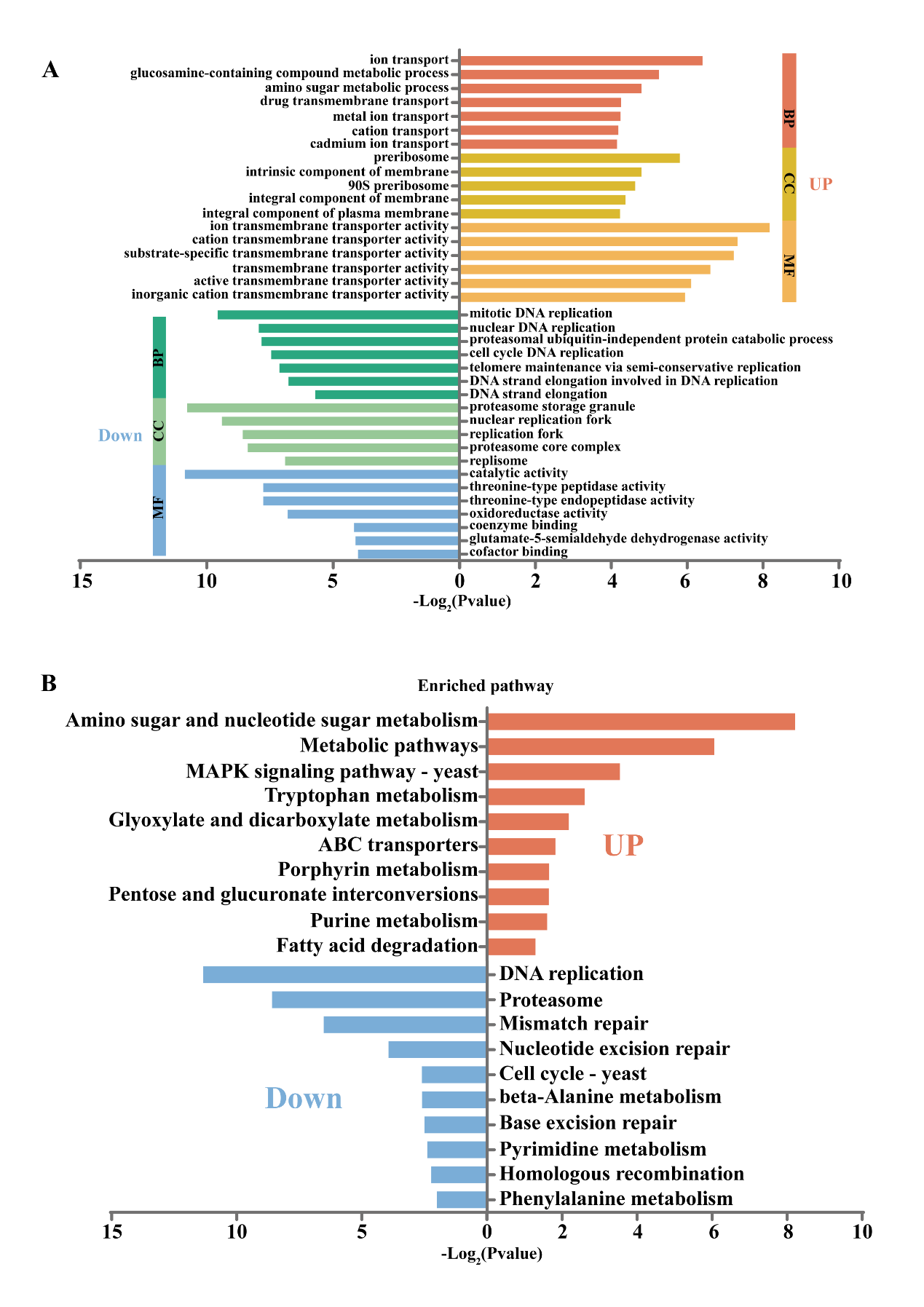


**Figure S8**

Analysis of differentially expressed genes in Δ*CpZap1* compared to KU80. A: GO-based enrichment analysis of DEGs in terms of biological process (BP), cell component (CC) and molecular function (MF). B: KEGG pathway enrichment analysis of DEGs.
